# Lysophosphatidic Acid Receptor 4 Is Transiently Expressed during Cardiac Differentiation and Critical for Repair of the Damaged Heart

**DOI:** 10.1016/j.ymthe.2020.11.004

**Published:** 2020-11-05

**Authors:** Jin-Woo Lee, Choon-Soo Lee, Yong-Rim Ryu, Jaewon Lee, HyunJu Son, Hyun-Jai Cho, Hyo-Soo Kim

**Affiliations:** 1 Strategic Center of Cell & Bio Therapy, Seoul National University Hospital, Seoul 03080, Republic of Korea; 2 Division of Cardiology, Department of Internal Medicine, Seoul National University Hospital, Seoul 03080, Republic of Korea; 3 Department of Molecular Medicine and Biopharmaceutical Sciences, Graduate School of Convergence Science and Technology and College of Medicine or College of Pharmacy, Seoul National University, Seoul 03080, Republic of Korea

**Keywords:** cell therapy, differentiation, GPCR, G protein-coupled receptor, myocardial infarction, stem cells

## Abstract

Efficient differentiation of pluripotent stem cells (PSCs) into cardiac cells is essential for the development of new therapeutic modalities to repair damaged heart tissue. We identified a novel cell surface marker, the G protein-coupled receptor lysophosphatidic acid receptor 4 (LPAR4), specific to cardiac progenitor cells (CPCs) and determined its functional significance and therapeutic potential. During *in vitro* differentiation of mouse and human PSCs toward cardiac lineage, LPAR4 expression peaked after 3−7 days of differentiation in cardiac progenitors and then declined. *In vivo*, LPAR4 was specifically expressed in the early stage of embryonal heart development, and as development progressed, LPAR4 expression decreased and was non-specifically distributed. We identified the effective agonist octadecenyl phosphate and a p38 MAPK blocker as the downstream signal blocker. Sequential stimulation and inhibition of LPAR4 using these agents enhanced the *in vitro* efficiency of cardiac differentiation from mouse and human PSCs. Importantly, *in vivo*, this sequential stimulation and inhibition of LPAR4 reduced the infarct size and rescued heart dysfunction in mice. In conclusion, LPAR4 is a novel CPC marker transiently expressed only in heart during embryo development. Modulation of LPAR4-positive cells may be a promising strategy for repairing myocardium after myocardial infarction.

## Graphical Abstract

**Figure:**
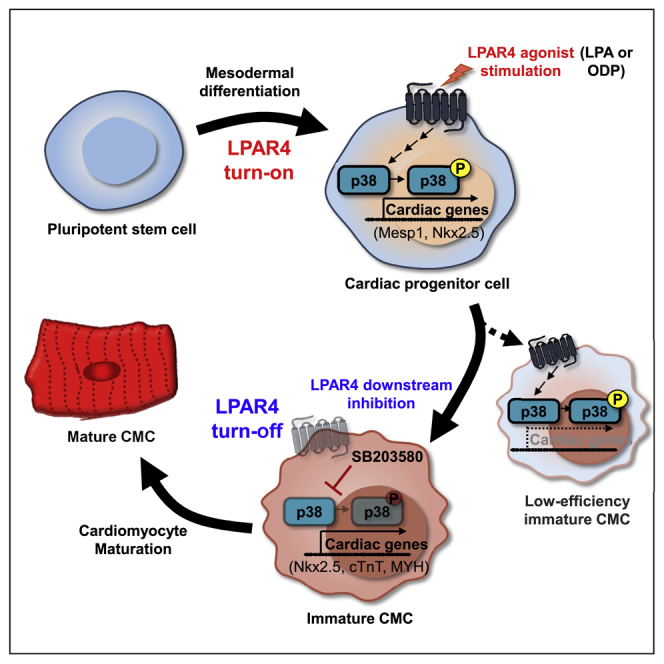

## Teaser

To effectively achieve cardiac differentiation from pluripotent stem cells, the discovery of new markers is essential. Lee et al. discovered a new cardiac progenitor cell marker, LPAR4, and its modulation showed functional significance during cardiac differentiation, providing new insight into cell-free cardiac repair by modulating LPAR4-positive cells in the heart.

## Introduction

The precise manipulation of embryonic stem cells (ESCs)/induced pluripotent stem cells (iPSCs) and the understanding of the characteristics of adult cardiac progenitor cells (CPCs) are essential for clinical applications.^1^, ^2^, ^3^ Cell-based therapy shows great potential for several clinical applications, particularly for tissue repair, including heart repair.^4^, ^5^, ^6^ However, its application to the regeneration of the injured cardiac tissue is limited by two major issues, i.e., the requirement to induce efficient lineage-specific stem cell differentiation^7^, ^8^, ^9^, ^10^ and the need to deliver CPCs or immature cardiomyocytes (CMCs) efficiently to the damaged heart.^5^^,^^11^^,^^12^ The identification of lineage-specific markers for CPCs could help the development of methods to drive CMC differentiation. However, even if cardiac differentiation is achieved, the effective delivery of CPCs or CMCs to the injured heart remains a substantial challenge.

To solve these issues, we searched for novel markers that specify cardiac lineage, using microarray analysis of four cell populations that differ in terms of the degree of enrichment of cardiac progenitors during differentiation of ESCs/iPSCs toward CMCs. We found that the G-protein-coupled receptor (GPCR)^13^ lysophosphatidic acid receptor 4 (LPAR4) is a strong candidate. Unlike well-known CPC markers, this newly discovered CPC marker is expressed on the cell surface and can regulate cardiac differentiation signals, enabling enrichment of CMCs. Another significant advantage of this novel CPC marker is that it can be used to characterize the function of the marker *in vivo* in mouse disease models since it is expressed in both mice and humans. The effectiveness of LPAR4 as a cardiac progenitor-specific marker and its functions were further evaluated based on its spatiotemporal expression patterns in the mouse heart during development and cardiac differentiation. Moreover, we confirmed the efficiency of cardiac differentiation with cardiac lineage markers through real-time PCR and fluorescence-activated cell sorting (FACS) analysis under various conditions to modify LPAR4 signaling using a combination of agonists, antagonists, and critical downstream signaling molecules. Besides, we used a mouse myocardial infarction (MI) model to highlight the concept of cell-free regeneration therapy with the optimal protocol to modulate the signaling of LPAR4.

## Results

### Identification of LPAR4 as a Cardiac Progenitor-Specific Marker during Differentiation of ESCs/iPSCs

**Figure 1:**
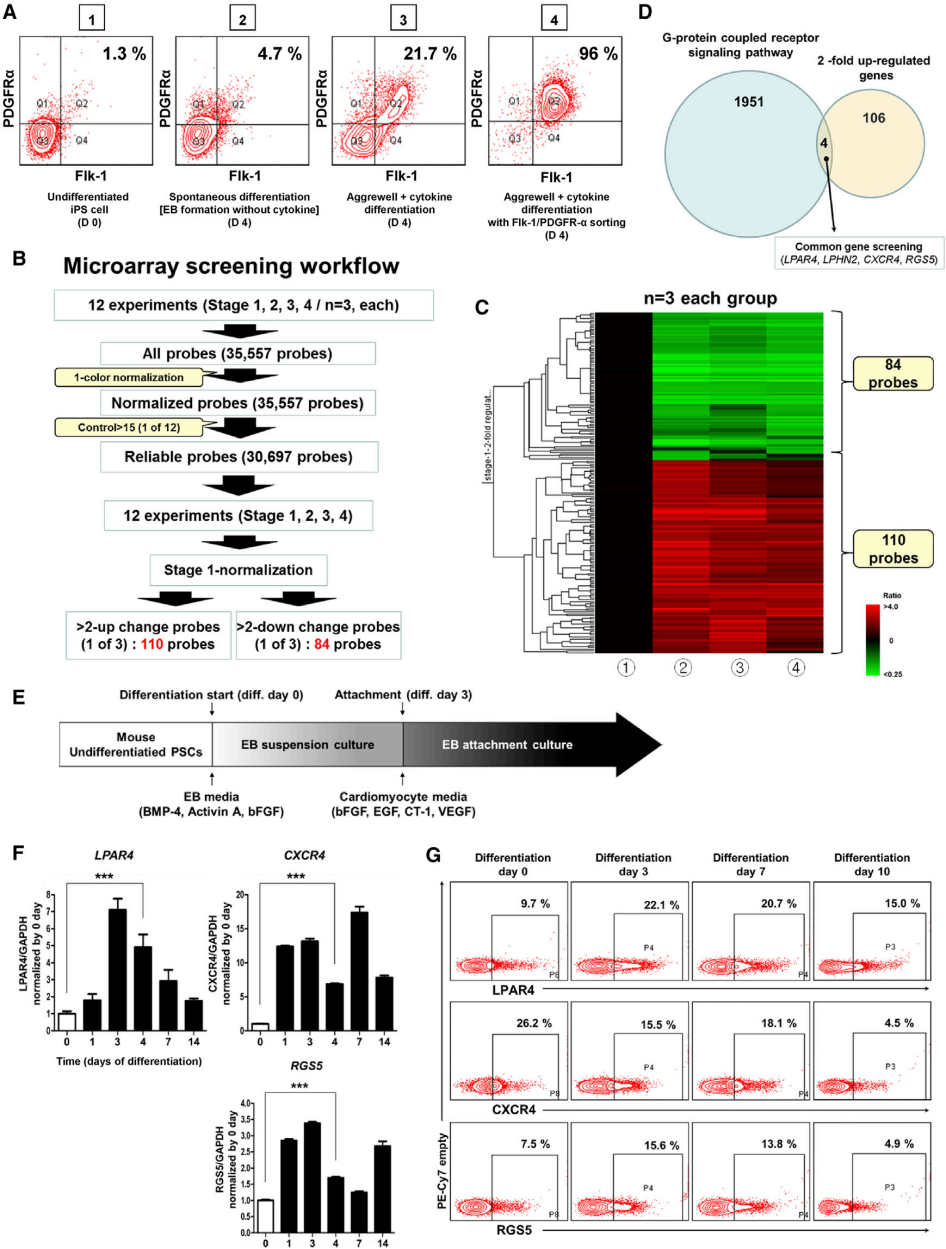
Identification and Expression of a New Cardiac Progenitor-Specific Marker (A) FACS analysis with the well-known cardiac progenitor markers Flk-1 and PDGFRα in undifferentiated induced pluripotent stem cells (iPSCs, group 1) and three cell lines at different stages of day 4-differentiation: group 2, spontaneously differentiated cells; group 3, cells differentiated by the established cardiac differentiation protocol; group 4, cells differentiated by the established cardiac differentiation protocol and sorted by Flk-1 and PDGFRα. (B) Microarray screening workflow for the four cell groups (n = 3). (C) Heatmap data from the microarray screening workflow. (D) Four candidate markers showing 2-fold upregulated expression are all G-protein–coupled receptors (GPCR): lysophosphatidic acid receptor 4 (*LPAR4*), Latrophilin 2 (*LPHN2*), chemokine (C-X-C motif) receptor 4 (*CXCR4*), and a regulator of G-protein signaling 5 (*RGS5*). (E) Schematic representation of the established cardiac differentiation protocol. (F) The real-time PCR analysis of the mRNA expression levels of the three candidates during cardiac differentiation. Error bars represent SEM, ∗∗∗p < 0.001, unpaired t test. n = 3 biological replicates. (G) FACS analysis of the protein expression levels of the three candidates during cardiac differentiation. All experiments were conducted at least in triplicate.

To identify a novel CPC-specific marker, we performed a microarray analysis using iPSCs^14^ at four different stages during differentiation. Figure 1A demonstrates flow cytometric analysis using the well-known cardiac lineage marker, fetal liver kinase 1 (Flk-1), and platelet-derived growth factor receptor alpha (PDGFRα)^15^ on day 4 of mouse iPSC differentiation into the cardiac lineage. Figure 1A shows (1) undifferentiated iPSCs, (2) spontaneously differentiated cells at day 4, (3) cells differentiated under the established cardiac differentiation protocol^16^ at day 4, and (4) cells cultured under the established cardiac differentiation protocol and sorted, at day 4, according to the cardiac lineage markers Flk-1 and PDGFRα. The detailed and optimized protocol used for the differentiation of iPSCs into the cardiac lineage is schematically presented in Figure 1E. Based on the microarray results, we selected genes that were upregulated by at least 2-fold as compared to undifferentiated iPSCs and the other three cell populations (Figures 1B and 1C). When we analyzed the microarray data by normalizing (1) the undifferentiated iPSCs as standard, we found 110 genes that were upregulated in cell stages (2), (3), and (4) on day 4. Since we were interested in identifying new surface markers, we looked for GPCR genes among the 110 upregulated genes, and discovered four genes: *LPAR4*, Latrophilin-2 (*LPHN2*), chemokine (C-X-C motif), receptor 4 (*CXCR4*), and the regulator of G-protein signaling 5 (*RGS5*; Figure 1D). During mouse cardiac differentiation from undifferentiated pluripotent stem cells (PSCs), LPAR4 mRNA and protein levels were expressed transiently. In particular, the expression of LPAR4 peaked between differentiation days 3 and 7 and then immediately disappeared. On differentiation day 14, LPAR4 mRNA and protein expression levels, as determined by qPCR and FACS, respectively, declined and were similar to those of the undifferentiated PSCs (Figures 1F and 1G). LPHN2 was published as a novel cardiac lineage marker that is expressed when PSCs differentiate into CPCs and maintain the expression until CMC differentiation.^16^ The expression pattern of CXCR4 or RGS5 is different from that of LPAR4, which fluctuated by peaking at day 4 and gradually decreasing during cardiac differentiation (Figures 1F and 1G). The LPAR4 mRNA and protein-expression pattern in the human iPSC line also showed transient expression similar to the pattern observed in the mouse PSC line (Figures S1A and S1B).

### Transient Expression Pattern of LPAR4 during Differentiation and Development

**Figure 2:**
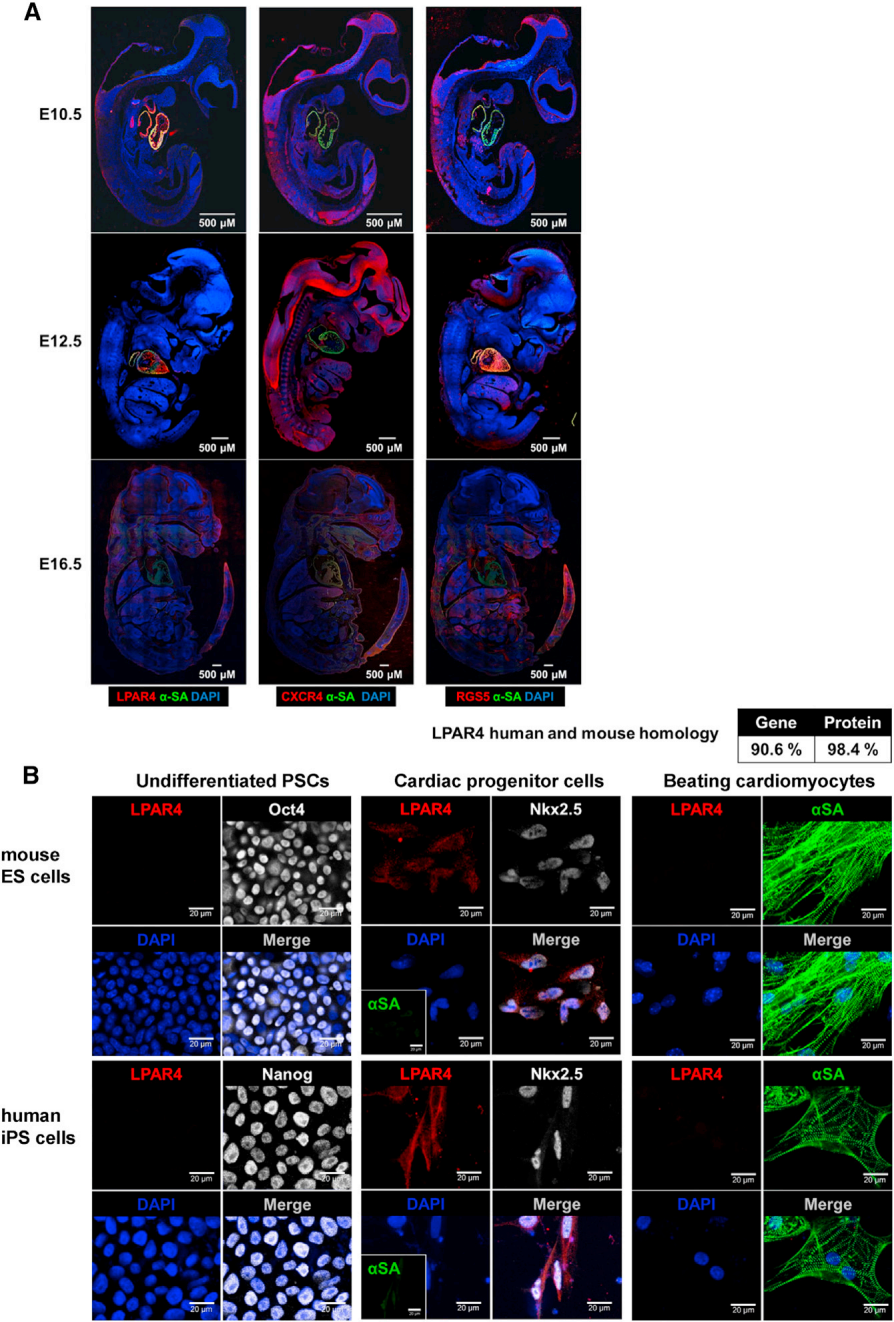
Transient Expression Pattern of LPAR4 (A) Immunofluorescence analysis during development at embryonic day 10.5, 12.5, and 16.5 (E10.5, E12.5, and E16.5). Red, CXCR4, RGS5, and LPAR4; green, αSA; DAPI, nuclei. Scale bar, 500 μm. (B) Immunofluorescence (IF) analysis of the correlation between representative cardiac progenitor markers and LPAR4 during differentiation from mouse ESCs and human iPSCs to CMCs. Red, LPAR4; green, αSA; white, Oct4, Nanog, and Nkx2.5; DAPI, nuclei. Scale bar, 20 μm. All experiments were conducted at least in triplicate.

The transient expression pattern of LPAR4 *in vitro* paralleled the observed expression pattern during mouse embryonic development. When we compared the heart-specific expression of three candidates, LPAR4, CXCR4, and RGS5, during mouse embryo development (Figure 2A), LPAR4 exhibited the most robust heart-specific expression at embryonic day 12.5 of development and significantly decreased at embryonic day 16.5, which represented transient expression pattern. Furthermore, RGS5 is not a GPCR and was excluded because it is expressed intracellularly rather than on the cell surface. Therefore, we further focused on LPAR4 as a useful surface marker of CPCs. We evaluated the mRNA expression of other members of the LPA receptor family^17^^,^^18^ during differentiation toward a cardiac lineage and found no significant change of expression in these family members, except for LPAR4 (Figure S2). Furthermore, immunostaining analysis confirmed that LPAR4 is transiently expressed in CPCs or early CMCs during differentiation from the undifferentiated stem cells at the protein level. ESCs and mature CMCs did not express LPAR4, whereas the cardiac progenitor state of immature cardiac CMCs expressed LPAR4. Transient cardiac-specific expression pattern of LPAR4 during differentiation was confirmed, not only in mouse ESCs, but also in human iPSCs (Figure 2B). LPAR4 gene and protein homologies between mice and humans were 90.6% and 98.4%, respectively (Figure 2B, upper panel). Applying the cardiac differentiation protocol to mouse ESCs, we compared LPAR4 expression with other cardiac progenitor markers, Flk-1 and PDGFRα, and found a very high correlation between expressions of LPAR4 and other cardiac progenitor markers (Figure S3). When we sorted cells depending on LPAR4 expression after 3 days of differentiation from mouse iPSCs toward CMCs, we could significantly enrich cardiac lineage cells in the LPAR4-positive cell population and exclude cardiac lineage cells from the LPAR4-negative one (Figure S4). LPAR4 is a cell surface marker transiently expressed during cardiac differentiation. Although the LPAR4 positive and negative cells were isolated and subjected to the cardiac differentiation protocol, only the LPAR4 positive cells differentiated into the cardiac lineage, indicating that LPAR4 is an essential protein for cardiac differentiation and CMC enrichment.

### Sequential Stimulation and Inhibition of LPAR4 Increases the Efficiency of Cardiac Differentiation from ESCs/iPSCs

**Figure 3:**
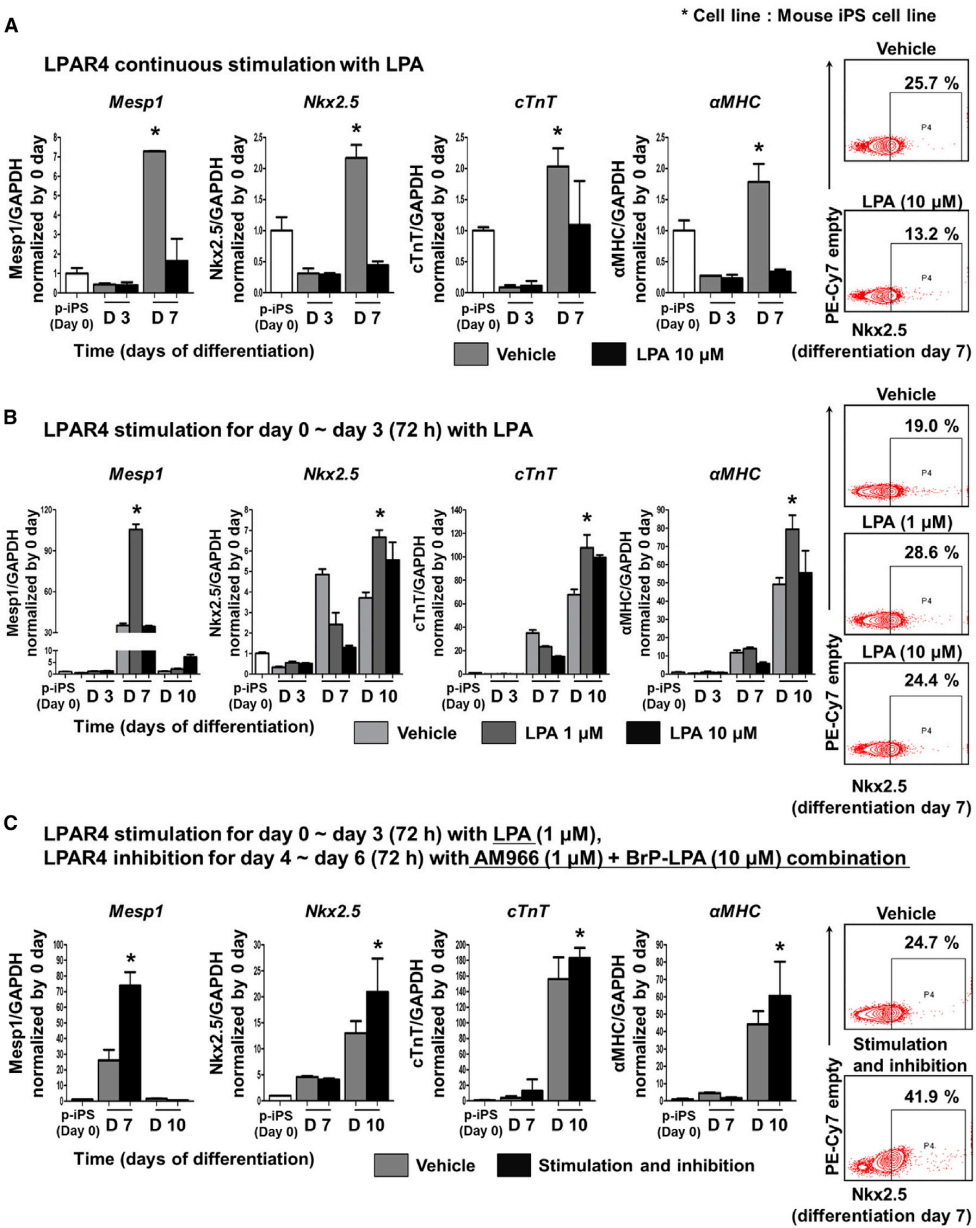
Protocol to Improve Cardiac Differentiation by LPAR4 Regulation (A) Real-time PCR and FACS analyses confirmed the expression of well-known cardiac progenitor markers when LPAR4 was continuously stimulated by treatment with 10 μM LPA by the established cardiac differentiation protocol. Real-time PCR analysis at cardiac differentiation day 3 and day 7, FACS analysis at day 7. (B) LPA stimulation during the early differentiation stage of the established cardiac differentiation protocol. LPAR4 was stimulated by 1 μM and 10 μM LPA for 3 days, and the results were compared to those of the untreated group. (C) LPAR4 was stimulated by 1 μM LPA for the first 3 days of differentiation and then inhibited by a combination of the antagonists AM966 and BrP-LPA for the next 3 days, and the results were compared with those of the untreated group. Statistical analyses were performed using one-way ANOVA (Newman-Keuls). ∗p < 0.01. All experiments were conducted at least in triplicate.

To validate the use of LPAR4 as a cardiac differentiation marker and its potential for stem cell therapy, we evaluated the cardiac differentiation efficiency after the stimulation or inhibition of LPAR4 considering the transient expression pattern of LPAR4 during differentiation from ESCs/iPSCs to CPCs. To stimulate LPAR4, we used lysophosphatidic acid (LPA), a representative agonist of the LPA receptor family. The continuous stimulation of LPAR4 with a high dose of LPA (10 μM) during cardiac differentiation, surprisingly decreased the cardiac differentiation efficiency as compared to the vehicle group, as confirmed by analyses of the mRNA and protein expression levels of cardiac genes^19^^,^^20^ performed by qPCR and FACS, respectively (Figure 3A). Next, we transiently stimulated cells with LPA at 1 μM and 10 μM only during the early stage of cardiac differentiation and observed significantly higher expression levels of cardiac genes as compared to the vehicle group; the lower dose of LPA (1 μM) was more effective than the higher dose of LPA (10 μM) in increasing gene expression levels (Figure 3B). Subsequently, we inhibited LPAR4 signaling using various LPAR4 antagonists to examine the effect on cardiac differentiation efficiency. Although an LPAR4 antagonist was required to suppress LPAR4 expression, none of the antagonists was specific for LPAR4, and various antagonists were tested. Among the tested compounds, AM966 and BrP-LPA affected cardiac differentiation. AM966^21^ only weakly blocks LPAR4 signaling, whereas BrP-LPA^18^^,^^22^^,^^23^ is a pan-LPA receptor family antagonist with high affinity for LPAR4. When the cells were treated with AM966 (1 μM) or BrP-LPA (10 μM) alone, the antagonist only slightly influenced cardiac differentiation; however, the cardiac differentiation efficiency was increased when treated with a combination of two antagonists (Figure S5). Finally, considering the transient expression pattern of LPAR4 during differentiation, we tried sequential stimulation (at the early stage) and then inhibition (at the late stage) of LPAR4 signaling. We sequentially stimulated LPAR4 with LPA (1 μM) for 3 days at the early cardiac differentiation phase and then inhibited LPAR4 with the antagonist combination of AM966 (1 μM) and BrP-LPA (10 μM) for the next 3 days. This sequential stimulation and inhibition of LPAR4 signaling pathway caused a significant increase in the expression of cardiac genes at both the mRNA and protein levels (Figure 3C). The same results were obtained using other mouse ESC lines (Figure S6).

Taken together, during the cardiac differentiation process, the stimulation of LPAR4 at the early stage with the low concentration of LPA but not with the high concentration, increases the efficiency of differentiation. Furthermore, when LPAR4 is stimulated at the early stage and then inhibited at the late stage by the antagonist combination, differentiation efficiency is much improved compared with LPA stimulation only. The regulation of LPAR4 signaling is a useful strategy to facilitate cardiac differentiation from pluripotent stem cells.

### Downstream Signaling Pathway of LPAR4: p38 MAPK

**Figure 4:**
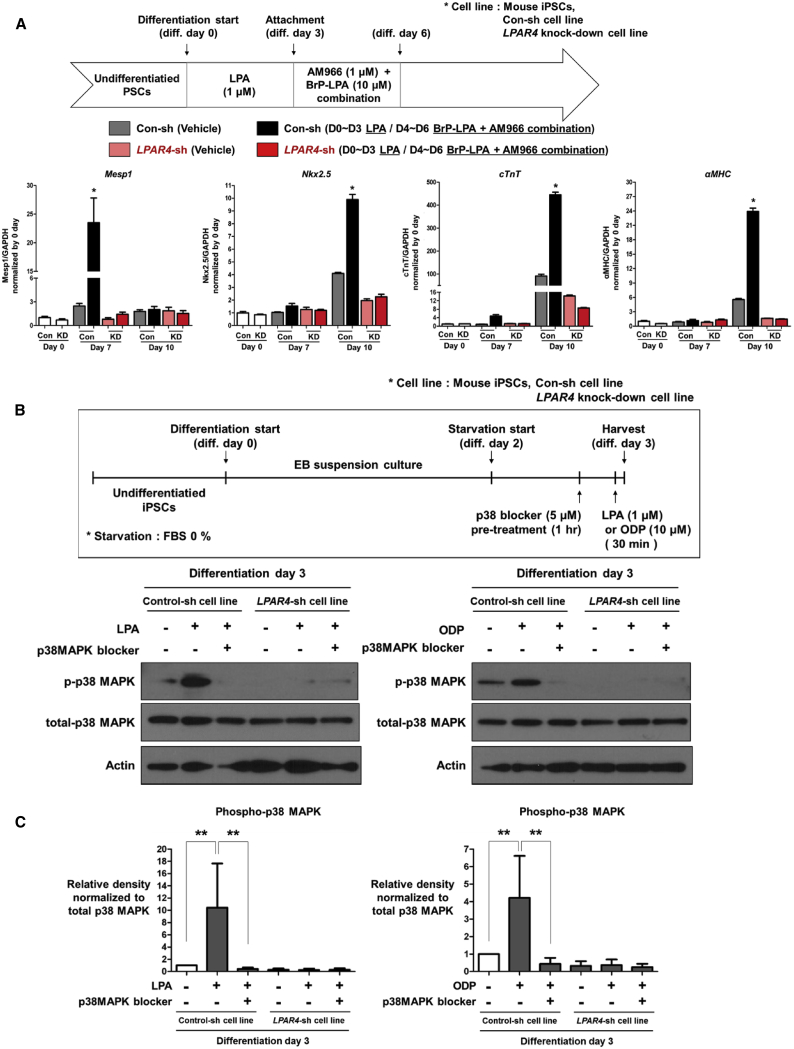
Identification of LPAR4 Downstream Signaling Molecules (A) Expression of cardiac progenitor markers in the *LPAR4*-knockdown cell line and control cell line by real-time PCR at cardiac differentiation day 7 and day 10; the *LPAR4*-knockdown cell line did not differentiate into CMCs. Statistical analyses were performed using one-way ANOVA (Newman–Keuls). ∗p < 0.01. (B) The effects of LPA and ODP were confirmed by western blotting in the control cell line and *LPAR4*-knockdown cell line at cardiac differentiation day 3. Both cell lines were starved for 1 day and treated with LPA for 30 min. The cells were treated with p38 MAPK 30 min before LPA treatment. All experiments were conducted at least in triplicate. (C) Quantification of western blot from Figure 4B. Error bars represent the mean of four independent experiments. ∗p < 0.01.

To demonstrate that LPAR4 is an essential molecule for cardiac differentiation, we transduced iPSCs with an *LPAR4*-knockdown lentiviral particle (Sigma-Aldrich, TRCN0000026398) to produce an *LPAR4*-knockdown cell line (*LPAR4*-sh cell line; Figure S7). We then compared the cardiac differentiation ability of the *LPAR4*-sh cell line with that of a control-sh cell line established by transducing iPSCs with a random-sequence lentiviral particle that does not influence cardiac differentiation. The sequential stimulation and inhibition of LPAR4 increased the cardiac differentiation efficiency in the control-sh cell line, as confirmed by the expression of cardiac lineage markers, whereas there was no such increase in the *LPAR4*-sh cell line, with or without the agonist and antagonist combination (Figure 4A).

We then attempted to identify signaling molecules that mediate the effects of LPAR4 in the cardiac differentiation process. We examined the phosphorylation of representative MAPK pathway, which is increased by LPA stimulation, through western blot analysis. Among the MAPK pathway downstream signaling molecules, only p38 MAPK increased in phosphorylation following LPA stimulation (Figure S8). Thus, we examined the phosphorylation of p38 MAPK in the *LPAR4*-sh and control-sh cell lines using western blotting. The stimulation of LPAR4 with LPA increased the phosphorylation of p38 MAPK^24^, ^25^, ^26^ in the control-sh cells on cardiac differentiation day 3 (Figures 4B and 4C; Figure S8). Induction of p38 MAPK phosphorylation by LPA was obliterated in the *LPAR4*-sh cell line.

To further optimize the cardiac differentiation protocol, we applied the LPAR4-specific agonist, octadecenyl phosphate (ODP),^27^ to stimulate LPAR4 more specifically and in a robust manner (Figure S9). As the concentration of ODP increased, the cardiac differentiation efficiency gradually increased. The optimal concentration of ODP during cardiac differentiation was 10 μM; higher concentrations were too toxic to permit cell survival. ODP, like LPA, increased phosphorylation of p38 MAPK, which was obliterated in *LPAR4*-sh cell lines (Figure 4B). Therefore, the novel agonist ODP stimulates LPAR4 specifically and improves the efficiency of cardiac differentiation.

### Novel Protocol to Induce Differentiation of ESCs/iPSCs toward Cardiac Lineage: Sequential Stimulation and Inhibition of LPAR4 Signaling

**Figure 5:**
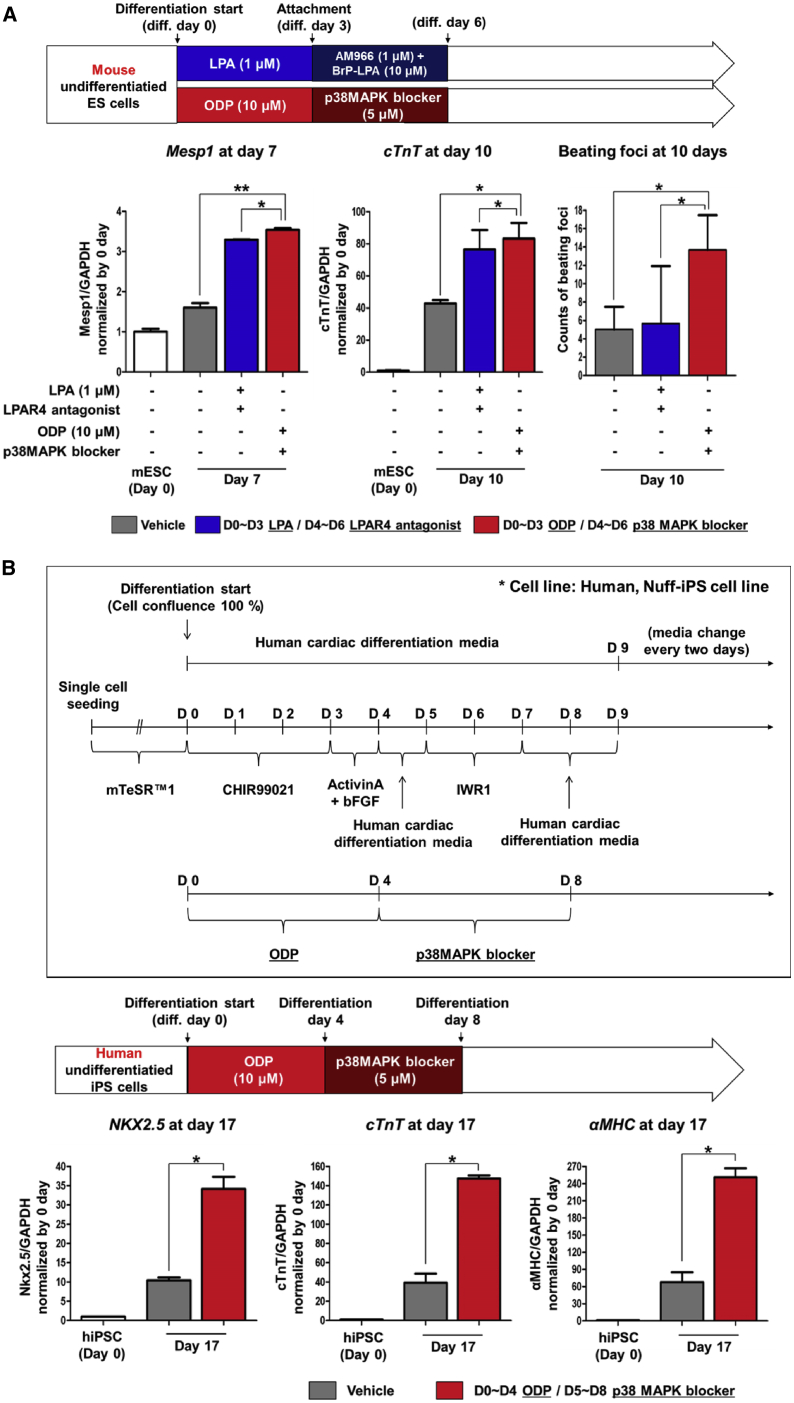
Cardiac Differentiation Protocol Using Sequential Stimulation and Then Inhibition of LPAR4 in Mouse ESCs and Human iPSCs (A) To maximize the cardiac differentiation efficiency, LPA or ODP was used as LPAR4 stimulants. LPAR4 antagonist combination (BrP-LPA and AM966) or p38 MAPK blocker was used as LPAR4 inhibitors. Mouse ESCs were differentiated into CMCs and confirmed using the cardiac lineage marker Mesp1 at cardiac differentiation day 7 and cTnT at cardiac differentiation day 10 by real-time PCR. Besides, beating CMCs were counted (displayed as a bar graph) to confirm the cardiac differentiation efficiency. (B) The human cardiac differentiation protocol is shown in a schematic figure (upper panel). The effects of ODP and the p38 MAPK blocker on the differentiation efficiency of human iPSCs into CMCs were evaluated. The cardiac lineage markers were analyzed by real-time PCR at cardiac differentiation day 17 (lower panel). Statistical analyses were performed using one-way ANOVA (Newman-Keuls). ∗∗∗p < 0.001. All experiments were conducted at least in triplicate.

To further increase the cardiac differentiation efficiency using LPAR4, we used an LPAR4-specific agonist and antagonist. To identify the most effective cardiac differentiation protocol, we compared LPA and ODP with each other as LPAR4 stimulants. The cardiac differentiation efficiency was significantly higher in the ODP-treated group than in the LPA-treated group, as confirmed by real-time PCR and estimates of beating foci (Figure S9). Next, we compared an LPAR4-antagonist combination (AM966 and BrP-LPA compounds) with a p38 MAPK inhibitor (SB203580 compound). We found that a blocker of p38 MAPK (SB203580), the LPAR4 downstream signaling molecule, was more effective than the combination of direct LPAR4 antagonists (AM966 and BrP-LPA) in inducing cardiac differentiation (Figure S10). To maximize cardiac differentiation, we designed a novel cardiac differentiation protocol of sequential treatment with the LPAR4-specific agonist ODP (10 μM) to stimulate LPAR4 and then with the p38 MAPK blocker (5 μM) to inhibit LPAR4 downstream signaling during differentiation. The number of beating CMCs from mouse ESCs was higher after ODP stimulation followed by p38 MAPK blocker treatment than in the untreated group (vehicle) or the sequential LPA-stimulated and antagonist combination-treated group (Figure 5A). Such sequential stimulation with ODP and inhibition with SB203580 compound in mouse ESC was also very useful in guiding “human iPSCs” to differentiate toward cardiac lineage as compared with control differentiation culture condition (Figure 5B). Moreover, confirmation of cTnT, a CMC structural protein, by FACS analysis, demonstrated that the group treated with the ODP and p38 MAPK blocker sequentially displayed higher cTnT positivity compared to the control differentiation group (Figure S11).

### LPAR4-Positive Cells from the Explant Culture of Mouse Heart

To test the feasibility of LPAR4-positive CPCs for clinical use, we tried to obtain these cells from mouse cardiac tissue. Based on our previous experience to obtain CPCs under the cardiosphere manufacturing protocol,^28^, ^29^, ^30^ we tried to obtain LPAR4-positive cells from the heart. We harvested healthy 3-week-old mouse hearts, which were chopped into similarly sized pieces and cultured *ex vivo* for 12 days.^28^^,^^31^ For explant culture, we used a 12-week-old mouse adult heart where LPAR4-positive cells were very rare. During several days of explant culture, cardiac progenitors, known as phase-bright cells, sprouted out from the explant center. Surprisingly, more than 90% of the sprouting cells or phase-bright cells were LPAR4-positive (Figure S12A). The explant culture was maintained for 12 days to harvest the greatest quantity of LPAR4-positive cells since LPAR4 expression is turned off afterward at the farthest point from the center of explant. We then applied the LPAR4-positive cells sprouting from the explant to the *in vitro* cardiac differentiation protocol used to differentiate ESCs/iPSCs into CMCs. We confirmed the efficacy of the protocol by observing that sequential stimulation and inhibition of LPAR4 signaling effectively activated the cardiac genes *Gata4*, *Isl1*, *Tbx5*,^32^ and *cTnT* (Figure S12B). *Gata4*, *Isl1*, *Tbx5*, and *cTnT* mRNA expression levels significantly increased when LPAR4 was sequentially stimulated and inhibited on *ex vivo* differentiation day 6. Immunostaining analysis showed that the α-SA protein expression level significantly increased in the group with sequential stimulation and inhibition of LPAR4 compared with that of the untreated group on *ex vivo* differentiation day 10 (Figure S12C). Although few LPAR4-positive cells are present in a 3-week-old mouse heart, these can be expanded during explant-culture and have the potential to differentiate into cardiac lineage cells, suggesting that LPAR4 may be an essential factor for the repair of adult heart after injury.

### Expression Pattern of LPAR4 in the Mouse Heart after Myocardial Infarction

Next, to confirm the therapeutic potential of LPAR4 *in vivo*, we examined its expression in healthy adult heart (7-week-old) and after myocardial infarction (MI).^29^ We compared LPAR4 mRNA and protein expression levels between healthy and MI hearts. Very low LPAR4 mRNA expression levels in the healthy myocardium significantly increased for 2 weeks after MI (Figures S13A and S13B). FACS analysis demonstrated that the LPAR4-positive cells in the single cell suspension of the heart specimens, significantly increased for 2 weeks after MI (Figure S13C). We analyzed the sequence of expression of LPAR4, Nkx2.5, and α-SA at the peri-infarct zone after MI (Figure S14A and S14B). At 3 days after MI, we observed LPAR4 but not NKX2.5, (Figure S14B, upper panel). Around 7 days after MI, Nkx2.5-positive cells appeared in the peri-infarct area, and these Nkx2.5-positive were also LPAR4-positive (Figure S14B, mid-panel). CPCs that were double-positive for LPAR4 and Nkx2.5 may have the potential to differentiate into CMCs but did not express the fully mature pattern of cytoskeleton or α-SA until day 14 (Figure S14B, lower panel). We observed that the number of LPAR4-positive cells increased for 2 weeks after MI in the mouse model. We also confirmed that LPAR4-positive cells progressively differentiate and express Nkx2.5 several days after MI in the peri-infarct zone.

### Effect of Sequential Stimulation and Inhibition of LPAR4 to Repair the Heart after MI in Mice

**Figure 6:**
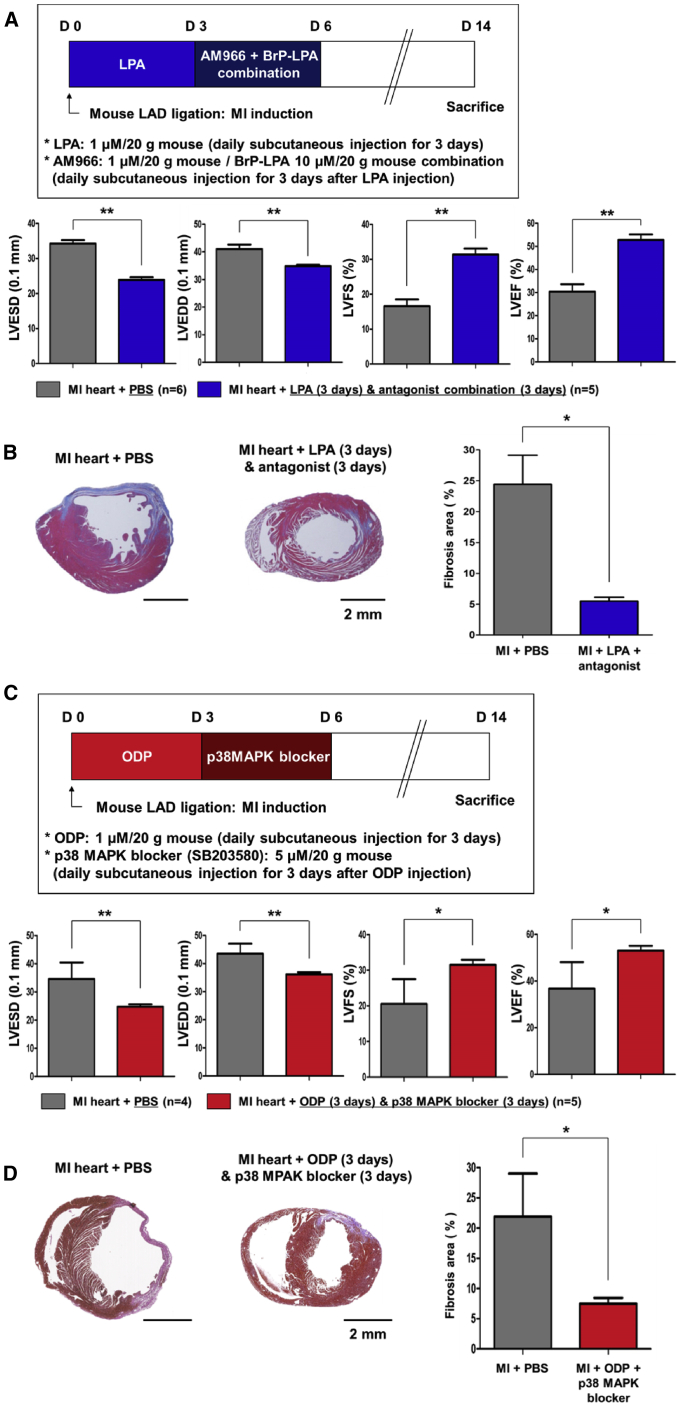
The Therapeutic Effect of Sequential Stimulation and then Inhibition of LPAR4 Signaling in a Mouse MI Model (A) Schematic representation of subcutaneously injected with the LPAR4 non-specific agonist LPA (1 μM/20 g) and antagonist combination (AM966 [1 μM/20 g] + BrP-LPA [10 μM/20 g]) in the mouse MI model. Echocardiography (MI + PBS group, n = 6; MI + LPA [3 days] + antagonist [3 days] group, n = 5). LVESD, left ventricular end-systolic diameters; LVEDD, left ventricular end-diastolic diameters; LVFS, left ventricular functional shortening; LVEF, left ventricular ejection fraction. (C) Schematic representation of treatment with the LPAR4-specific agonist ODP (10 μM/20 g) and specific downstream signaling molecule blocker (p38 MAPK blocker [SB203580; 5 μM/20 g]) in the mouse MI model. Echocardiography (MI + PBS group, n = 4; MI + ODP [3 days] + p38 MAPK blocker [3 days] group, n = 5). (B and D) The hearts were fixed, sectioned, and MT stained. The relative fibrotic area was measured using SABIA. Statistical analyses were performed using one-way ANOVA (Newman-Keuls). ∗∗∗p < 0.001. Scale bar, 2 mm.

We applied the established protocol of sequential stimulation and inhibition of LPAR4 signaling to repair the myocardium after infarction in mice. We first stimulated and inhibited LPAR4 using a sequential injection of the non-specific agonist LPA and non-specific antagonist combination. The LPA and antagonist combination was subcutaneously injected sequentially into the mouse MI model. We measured the degree of myocardial repair using echocardiography and histologic evaluation of fibrosis. The strategy of *in vivo* LPAR4 stimulation and inhibition in mice after MI, as shown in the schematic diagram in Figure 6A (upper panel), was the same as the strategy used *in vitro* during the differentiation from mouse ESCs/iPSCs to CMCs. Heart function was evaluated at 14 days after MI induction and was compared with the sham group. The pathologic left ventricle (LV) dilatation and contractile function of MI heart improved substantially in the group treated with LPA as compared with the control PBS group (Figure 6A, lower panel). Moreover, the group with sequential LPAR4 stimulation and inhibition showed enhanced heart function than the control PBS group (Figure 6A, lower panel). In the histologic analysis, the control PBS group showed large infarct and compensatory hypertrophy of LV, which was remarkably reduced in the group treated with sequential LPA and its antagonists (Figure 6B, left panel). Besides, the size of the fibrosis area examined by MT (Masson’s trichrome) staining decreased mainly in the group treated with sequential stimulation and inhibition of LPA signaling compared with the PBS group (Figure 6B, right panel).

Next, we tested the specific agonist ODP and p38 MAPK blocker in a mouse MI model to eliminate the off-target effects of the non-specific agonist and antagonist on various signaling molecules via the other members of the LPA receptor family (Figure 6C, upper panel). ODP and p38 MAPK blockers were subcutaneously injected sequentially into mice. The sequential stimulation and inhibition with ODP and the p38 MAPK blocker recovered the MI heart function to the level observed in sham hearts (Figure 6C, lower panel). The pathologic LV dilatation and fibrosis area were also significantly reduced in the group treated with sequential ODP and p38 MAPK blocker, as compared to PBS injection (Figure 6D). Based on these *in vivo* data, we confirmed that the protocol of sequential stimulating and then inhibition of LPAR4 signaling not only markedly increased CMC differentiation from ESC/iPSC but also boosted up post-infarction myocardial repair.

## Discussion

We describe for the first time that LPAR4 is a novel cardiac progenitor-specific cell surface marker of PSC differentiation and plays a vital role in the functional recovery of a damaged adult heart. For conventional CMC enrichment techniques, the marker-positive cells should be isolated utilizing an external surface protein method such as cell sorting. Since LPAR4 is a functional receptor as GPCR, downstream signals can be tuned using an agonist or antagonist to facilitate the maturation of CPCs, and ultimately to enrich the CMC population without cell damage.

During differentiation from PSC to CMC, LPAR4 is transiently expressed specifically in CPCs that appeared between Mesp1^33^ and Nkx2.5. Similar to Wnt,^34^ LPAR4 appears to be transiently expressed in the early stage of cardiac differentiation and then gradually disappears. Furthermore, during the embryonic heart development, LPAR4 is spatiotemporally and specifically expressed in the heart between E10.5 and E12.5, and then the expression of LPAR4 is broadly expressed in the whole body at E16.5.

In this study, we focused on the biphasic behavior of LPAR4 expression during differentiation of CPCs from PSCs and developed the two-phase protocol in which LPAR4 was stimulated and then inhibited. In mouse ESCs/iPSC differentiation into CMCs, the first phase was the progression of undifferentiated PSCs to the mesodermal lineage. The LPAR4 expression level was significantly turned on at the initial stage of differentiation. Stimulation of LPAR4 with their agonists during days 0 to 3 efficiently induced PSCs into the mesodermal lineage. In the second phase, we suppressed LPAR4 signaling using a downstream blocker after the initial stage of stimulation with agonists to effectively induce mesodermal lineage cells toward CPCs and CMCs.

For the early phase stimulation of LPAR4, we used two different types of LPAR4 agonist, LPA, and ODP. Although LPA simulates LPAR4 and improves the efficiency of cardiac differentiation, the affinity of LPA for LPAR4 was known to be weaker than that for other LPA receptor family. We conclude that a more specific agonist will be able to tune LPAR4 signaling selectively. Subsequently, we observed that the ODP stimulation showed higher efficiency than LPA stimulation (Figure S9). For the late phase inhibition of LPAR4, AM966, an LPAR1-specific antagonist, and BrP-LPA, were used to antagonize the entire LPA receptor family. The effect was negligible when either of the antagonists was applied individually. Despite this, when the two antagonists were administered simultaneously after the early phase (day 3~6), cardiac differentiation efficiency significantly increased. However, since the combination of AM966 and BrP-LPA is not an LPAR4-specific antagonist, we changed the strategy toward direct inhibition of downstream of LPAR4, p38 MAPK, using SB203580 compound after early phase (day 3~6). Our results showed that p38 MAPK, one of the MAPK, was the key LPAR4 downstream signaling molecule detected by western blotting (Figure 4B). The number of beating foci was significantly increased by the p38 MAPK blocker compared with a combination of non-specific antagonists (Figure 5A). Thus, we were able to establish an optimal protocol, the sequential stimulation of LPAR4 using specific agonists such as ODP and selective downstream inhibition of p38 MAPK. This optimized protocol could achieve the highest efficiency in the differentiation of mouse and human PSCs toward a cardiac lineage *in vitro*, as well as *in vivo* myocardium repair after infarction. The well-known downstream signaling of LPAR4 is the intracellular concentration of cAMP accumulation via Gs and adenylyl cyclase.^18^^,^^35^ Furthermore, the accumulated cAMP activates p38 MAPK.^36^ Our future studies will address the signaling pathway of LPAR4-Gs-cAMP-p38 MAPK.

Similar to the LPAR4 expression pattern in the cardiac differentiation process *in vitro*, LPAR4 was expressed in the mouse heart specifically at the early developmental stage. Another important *in vivo* finding suggesting a pathophysiologic role of LPAR4 was that the number of LPAR4-positive cells increased immediately after MI around the peri-infarct zone. Future studies should determine whether the LPAR4-positive cells found in the peri-infarct zone of adult mouse heart are resident stem/progenitor cells or cells infiltrated from other tissues such as bone marrow. In addition, we can infer several mechanistic actions of LPAR4 modulation resulting in MI repair, such as, re-activation of resident progenitors, differentiation of infiltrating cells, or immune modulation effects of those cells.^37^^,^^38^ In future studies, we aim to clarify the identity of LPAR4-positive cells which emerged at the peri-infarct zone of adult mouse heart, using the LPAR4-lineage tracing model. Nevertheless, our established protocol of sequential stimulation and inhibition of LPAR4 signaling could efficiently trigger cardiac tissue repair after MI, suggesting a possible imminent implementation in clinical practice. Various kinds of p38 MAPK blockers have already entered clinical trials, including in studies of inflammatory diseases.^39^ Thus, as LPAR4 signaling is transiently enhanced after MI, p38 MAPK blockers that inhibit LPAR4 signaling may be good candidates for cardio-protective medicine in the future. The main advantage of this strategy is that it relies on LPAR4-positive cells in the heart without requiring the injection of CPCs or the application of cell patches. LPAR4-positive cells appear during the acute phase after MI, and thus LPAR4 modulation alone can enhance the myocardial repair after MI.

Altogether, the results of our *in vitro* and *in vivo* experiments demonstrate that LPAR4 is a novel CPC marker transiently expressed only in the heart during embryo development. The fact that LPAR4 is a marker of CPCs and a GPCR, i.e., a functional membrane protein, has critical implications in cardiac repair. By targeting LPAR4, we can solve two important long-standing issues for the repair of the injured heart. As a CPC stage-specific marker, LPAR4 maximizes the ESC/iPSC-derived cardiac differentiation efficiency by sequential stimulation and inhibition. Also, a cell-free regeneration therapy can be realized using LPAR4-positive cells, which are already increased in the damaged heart, by sequential stimulation and inhibition of LPAR4 signaling using specific agonists and a downstream signaling blocker. Importantly, this strategy does not require the delivery of CPCs or CMCs. Additional experiments are needed for the development of LPAR4-specific agents (agonists and antagonists) that are safe for clinical applications. During development, LPAR4 expression level was only identified in the embryonic heart but was no longer heart-specific at E16.5. Future studies on LPAR4 expression in various tissues, as well as the heart, are also required.^40^

## Materials and Methods

We searched for novel CPC-specific marker using microarray analyses of four cell populations that were different from each other in the degree of enrichment with CPCs during differentiation from ESCs/iPSCs toward CMCs *in vitro*. We confirmed improvement in the efficiency of cardiac differentiation by the candidate marker identified in this analysis using various experimental techniques. The mRNA expression levels were determined by real-time PCR and FACS, immunofluorescence (IF), and western blotting were used to quantify protein expression levels. Microarray results are accessible at the GEO database (GEO: GSE83434)).

The methods are described in detail in the Supplemental Information.

## Supplemental Information

Document S1. Figures S1–S14, Table S1, and Supplemental Materials and Methods

Document S2. Article plus Supplemental Information
