# Supplementary material for: Lysophosphatidic Acid Receptor 4 Is Transiently Expressed during Cardiac Differentiation and Critical for Repair of the Damaged Heart
Source: Mol Ther. 2020 Nov 5;29(3):1151–63. doi: 10.1016/j.ymthe.2020.11.004 (PMC7934582; doi:10.1016/j.ymthe.2020.11.004)
Supplement: Document S1. Figures S1–S14, Table S1, and Supplemental Materials and Methods [file mmc1.pdf]

YMTHE, Volume *29*

Supplemental Information

Lysophosphatidic Acid Receptor 4 Is Transiently Expressed during Cardiac Differentiation and Critical for Repair of the Damaged Heart

Jin-Woo Lee, Choon-Soo Lee, Yong-Rim Ryu, Jaewon Lee, HyunJu Son, Hyun-Jai Cho, and Hyo-Soo Kim

Supplemental Information

Lysophosphatidic Acid Receptor 4 is transiently expressed during cardiac differentiation and critical for repair of the damaged heart

Jin-Woo Lee, Choon-Soo Lee, Yong-Rim Ryu, Jaewon Lee, HyunJu Son,

Hyun-Jai Cho, and Hyo-Soo Kim

Supplemental Figure 1

A

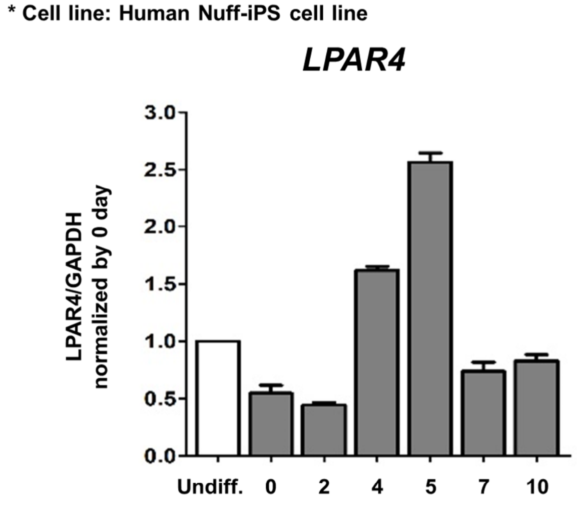

B

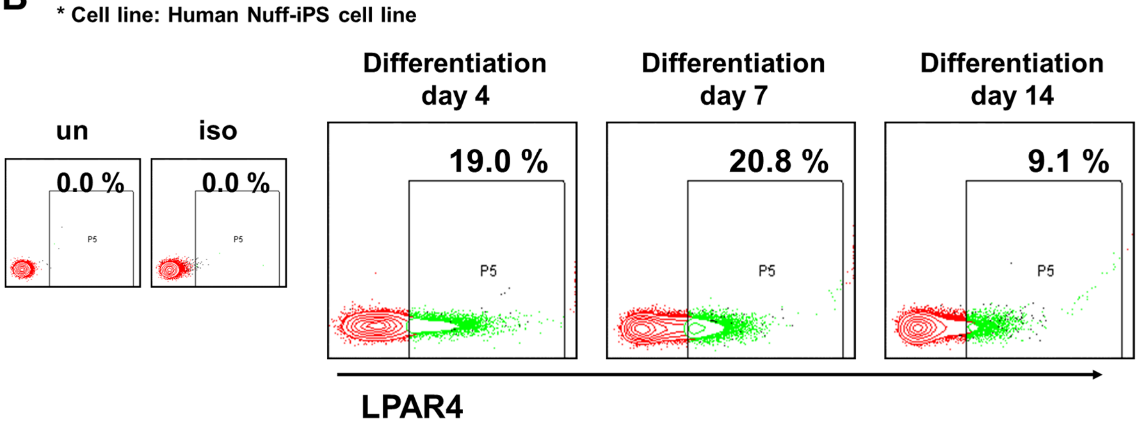

Supplemental Figure 1. Transient expression pattern of LPAR4 mRNA in human iPS cell line.

A During cardiac differentiation of human iPS cell line, the mRNA expression level of LPAR4 was confirmed by harvesting each cardiac differentiation day (undifferentiation state, differentiation day 0, day 2, day 4, day 5, day 7, and day 10). All experiments were conducted at least in triplicate.

B During cardiac differentiation of human iPS cell line, the protein expression level of LPAR4 was confirmed by FACS analysis (differentiation day 4, day 7, and day 10).

Supplemental Figure 2

< Lysophosphatidic acid receptor family >

Realtime-PCR

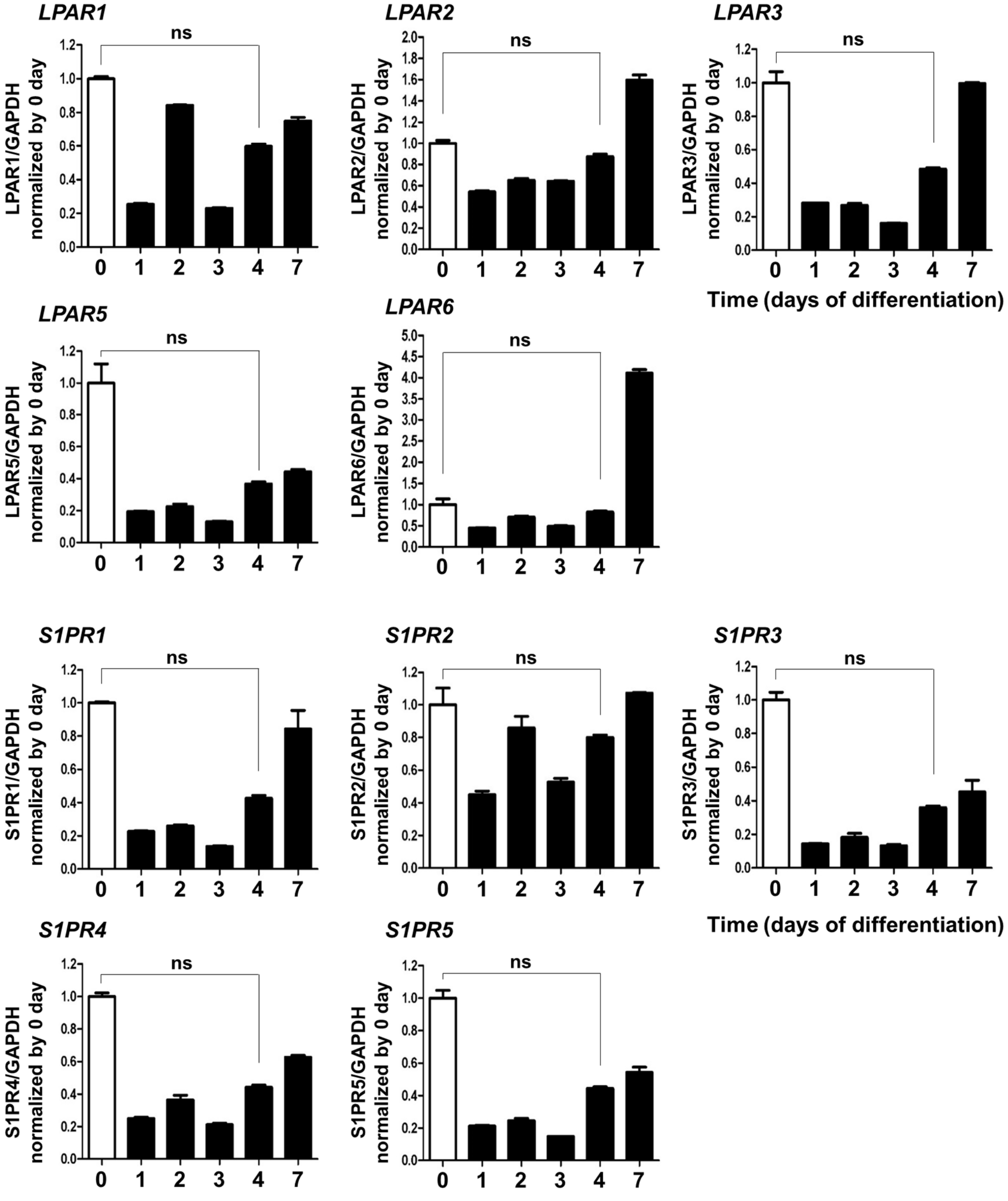

Supplemental Figure 2. Sequential expression pattern of members of the LPA receptor family during differentiation of pluripotent stem cells toward cardiomyocytes.

During cardiac differentiation, the mRNA expression pattern of *LPAR4* was confirmed to be different from that of other members of the LPA receptor family. In addition, the expression pattern of the sphingosine-1-phosphate receptor (S1PR) family, part of the lysophospholipid receptor family, was confirmed during the cardiac differentiation process. All experiments were conducted at least in triplicate. There were no significant differences in the LPA receptor family compared to differentiation day 0 with day 4. ns; not significant.

Supplemental Figure 3

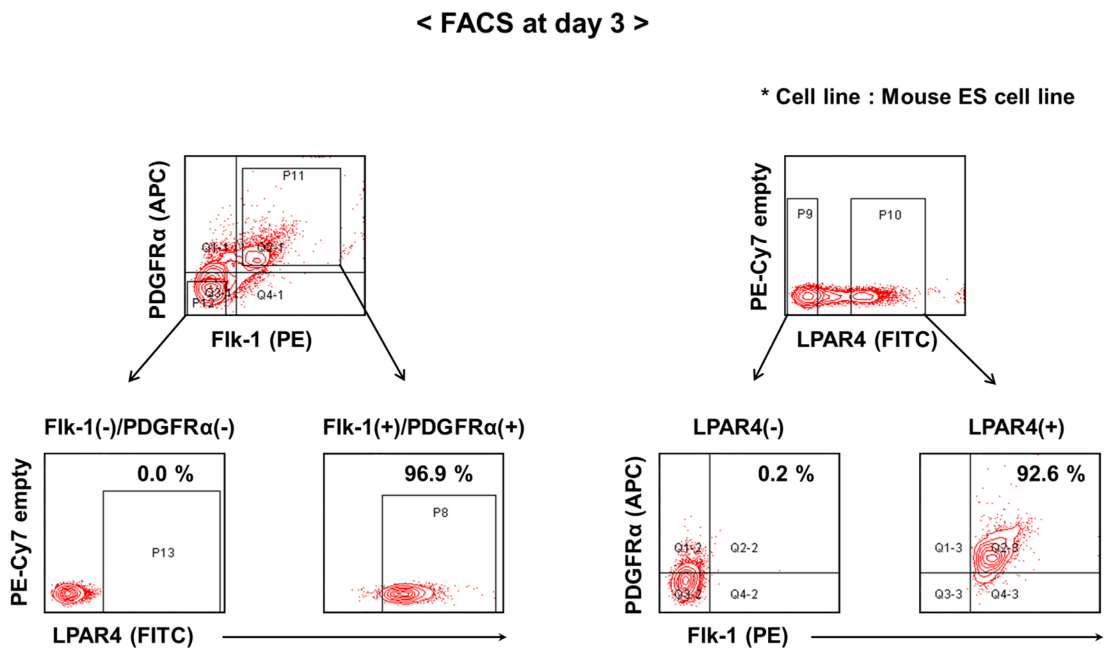

Supplemental Figure 3. Overlapping expression of LPAR4 with other cardiac progenitor marker such as double-positive expression of Flk-1 and PDGFRα.

Correlation of LPAR4 expression with that of well-known cardiac progenitor markers, Flk-1 and PDGFRα, during cardiac differentiation at day 3. All experiments were conducted at least in triplicate.

Supplemental Figure 4

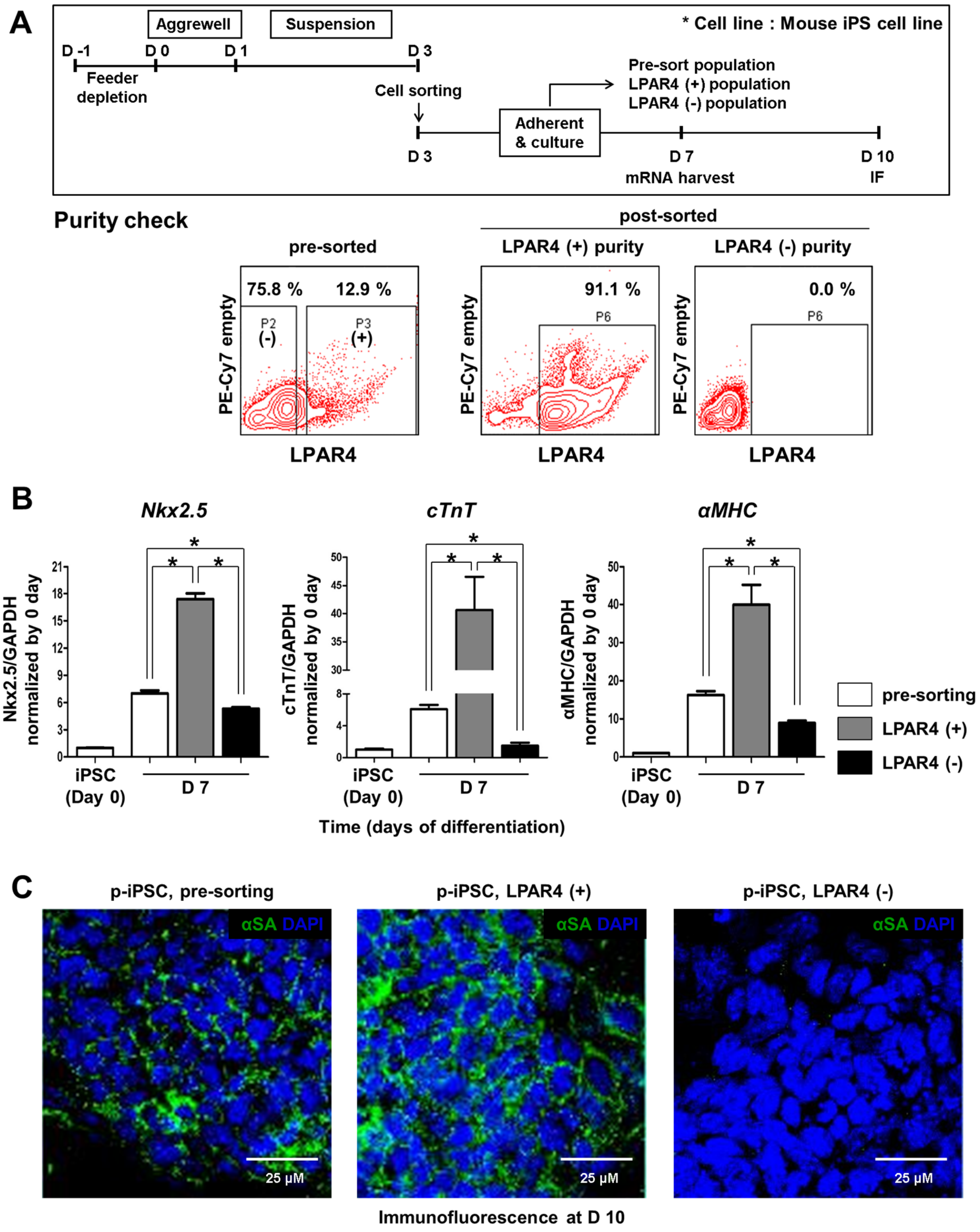

Supplemental Figure 4. Comparison of cardiac differentiation efficiency between LPAR4-positive cells versus LPAR4-negative ones.

A Scheme of cell sorting at cardiac differentiation day 3 using LPAR4 antibody and purity of LPAR4-positive and negative-populations after sorting and re-attachment culture under the established cardiac differentiation protocol.

B, C Cardiac differentiation efficiency of LPAR4-positive and -negative populations compared with that of the pre-sorted population analyzed by real-time PCR and immunofluorescence. Green-αSA, DAPI used for staining the nuclei. Bar, 25 μM. Statistical analyses were performed using one-way ANOVA (Newman–Keuls). *$P < 0.01$. All experiments were conducted at least in triplicate.

Supplemental Figure 5

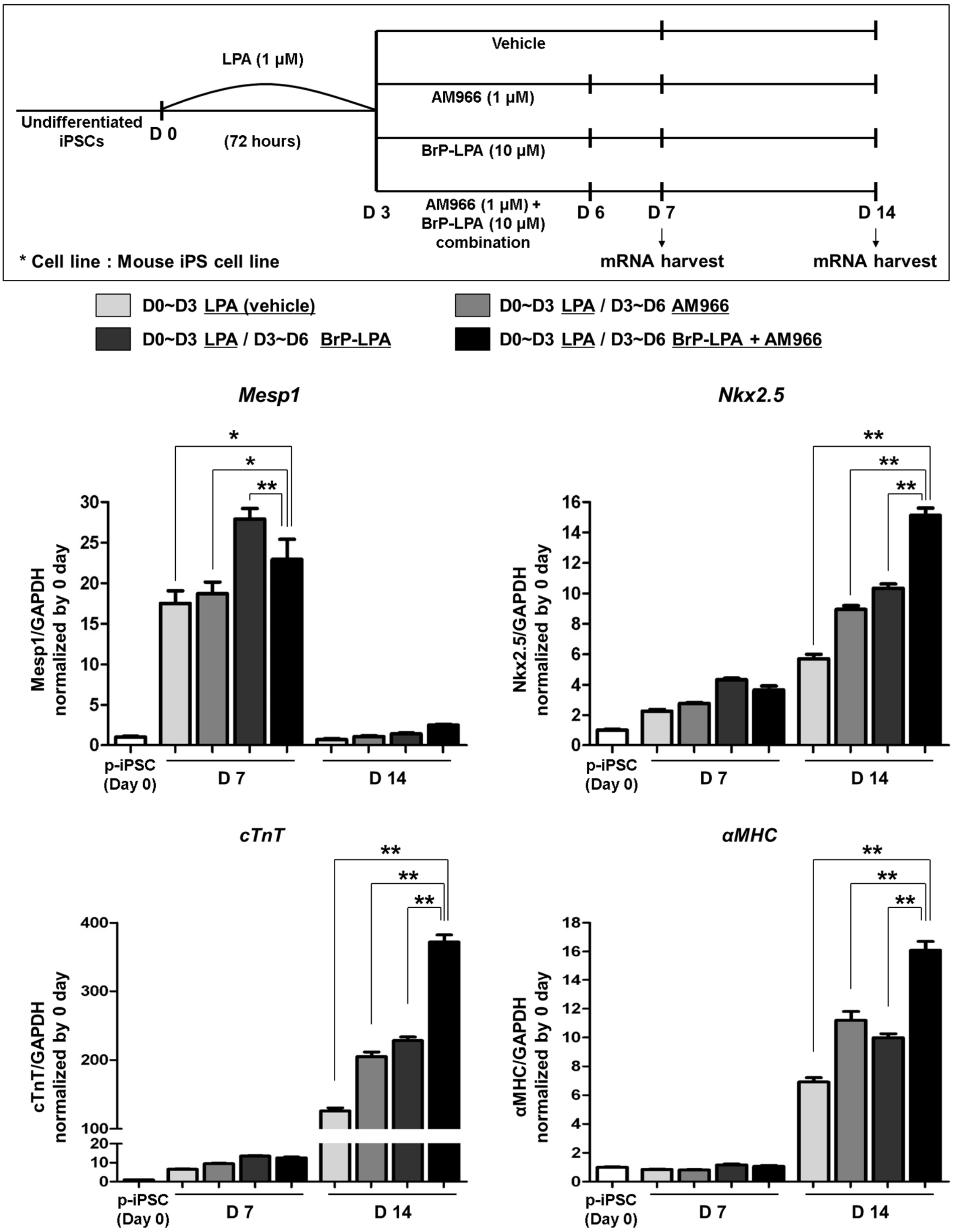

Supplemental Figure 5. Comparison of Antagonists for the most effective cardiac differentiation among AM966 and BrP-LPA.

After LPAR4 stimulation, LPAR4 was inhibited using AM966, BrP-LPA, and AM966 / BrP-LPA combination, which are well known as LPA receptor family antagonists, and cardiac differentiation efficiency was confirmed through mRNA levels of cardiac lineage markers respectively. Statistical analyses were performed using one-way ANOVA (Newman–Keuls). ***$P < 0.001$. All experiments were conducted at least in triplicate.

Supplemental Figure 6

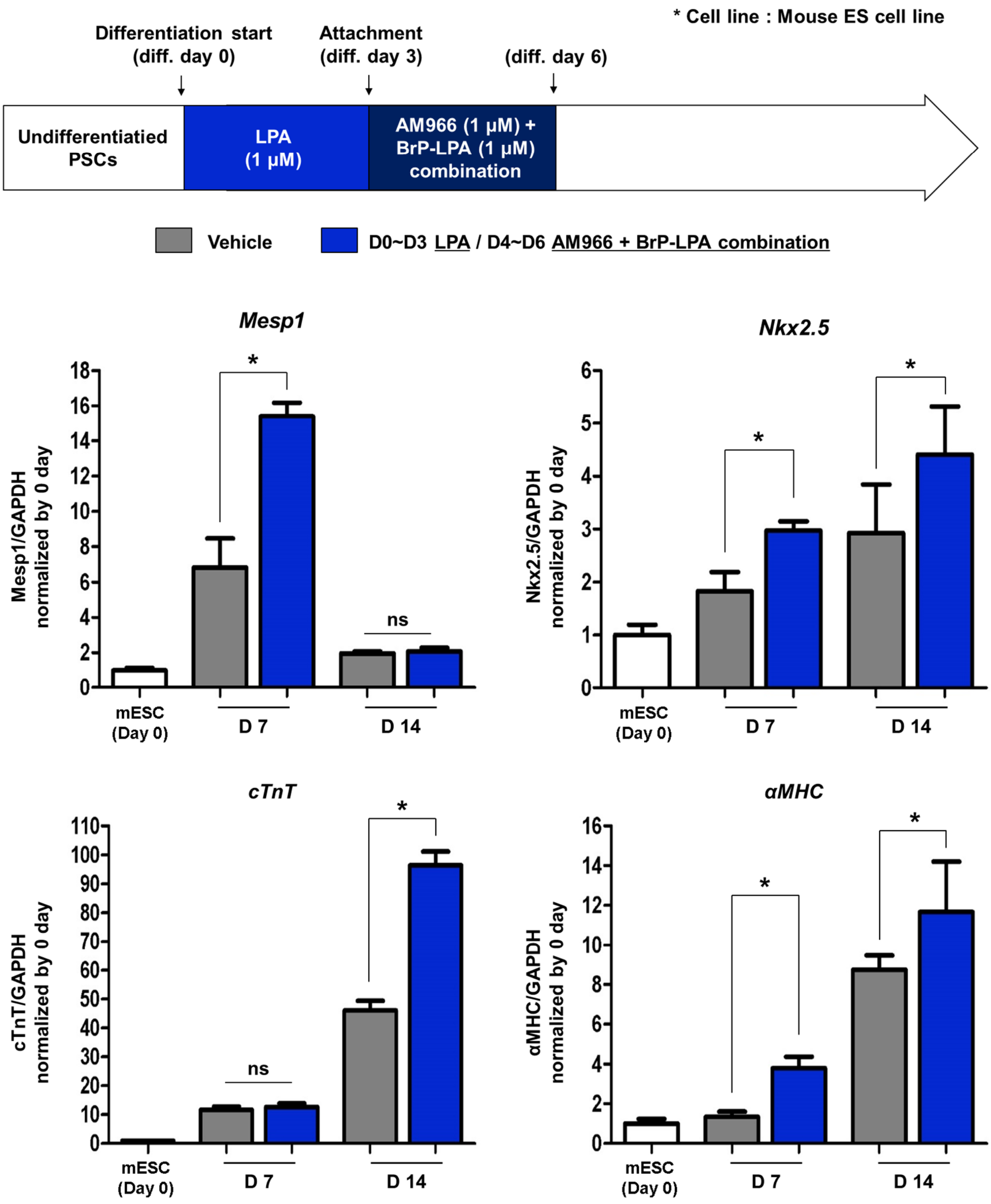

Supplemental Figure 6. Effect of sequential stimulation and inhibition of LPAR4 signaling using LPA and combination of antagonists, BrP-LPA and AM966, on cardiac differentiation using mouse ES cell line.

Schematic representation of cardiac differentiation efficiency in an embryonic stem cell (ESC) line (upper panel). Real-time PCR analysis with cardiac lineage markers at cardiac differentiation day 7 and 14 normalized by the mouse ESC line (lower panel). Statistical analyses were performed using one-way ANOVA (Newman–Keuls). *$p < 0.01$, ns: not significant. All experiments were conducted at least in triplicate.

Supplemental Figure 7

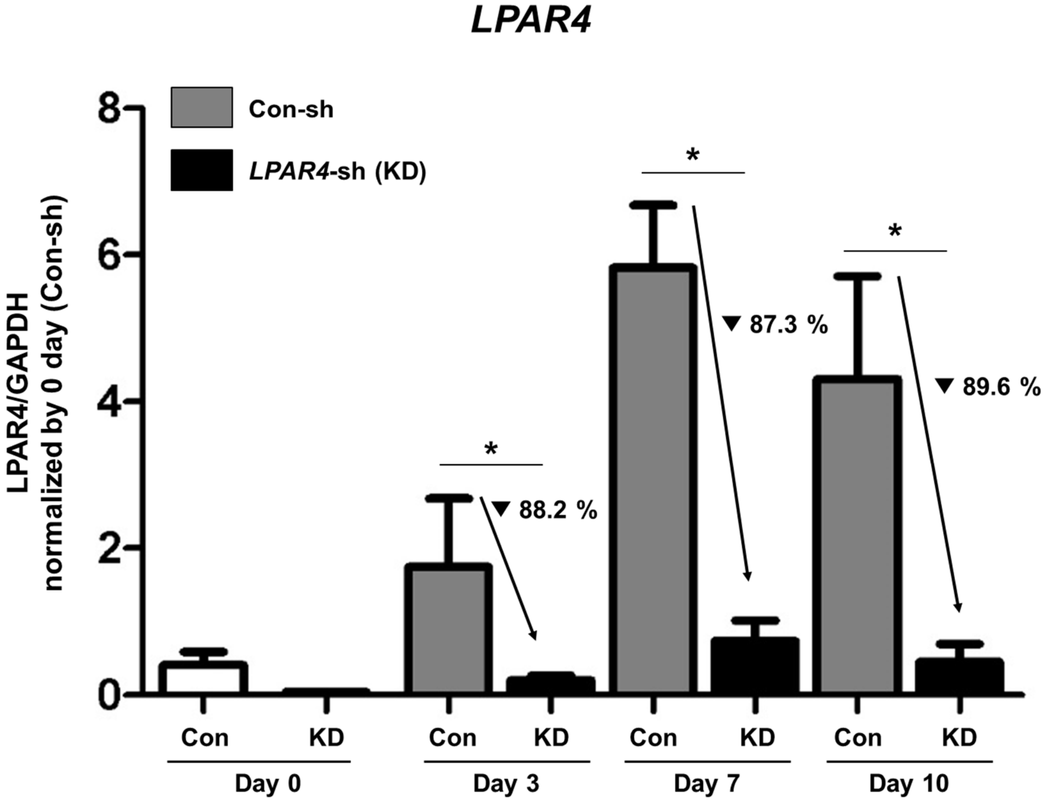

Supplemental Figure 7. LPAR4 expression in control cells versus *LPAR4*-knockdown cell line (*LPAR4*-sh).

The *LPAR4*-knockdown cell line was constructed by transfecting *LPAR4* knockdown lentiviral particles into iPSCs. Subsequently, Subsequently, *LPAR4* mRNA expression levels were compared with control cell lines (Con-sh). Statistical analyses were performed using one-way ANOVA (Newman–Keuls). *$P < 0.01$. All experiments were conducted at least in triplicate.

Supplemental Figure 8

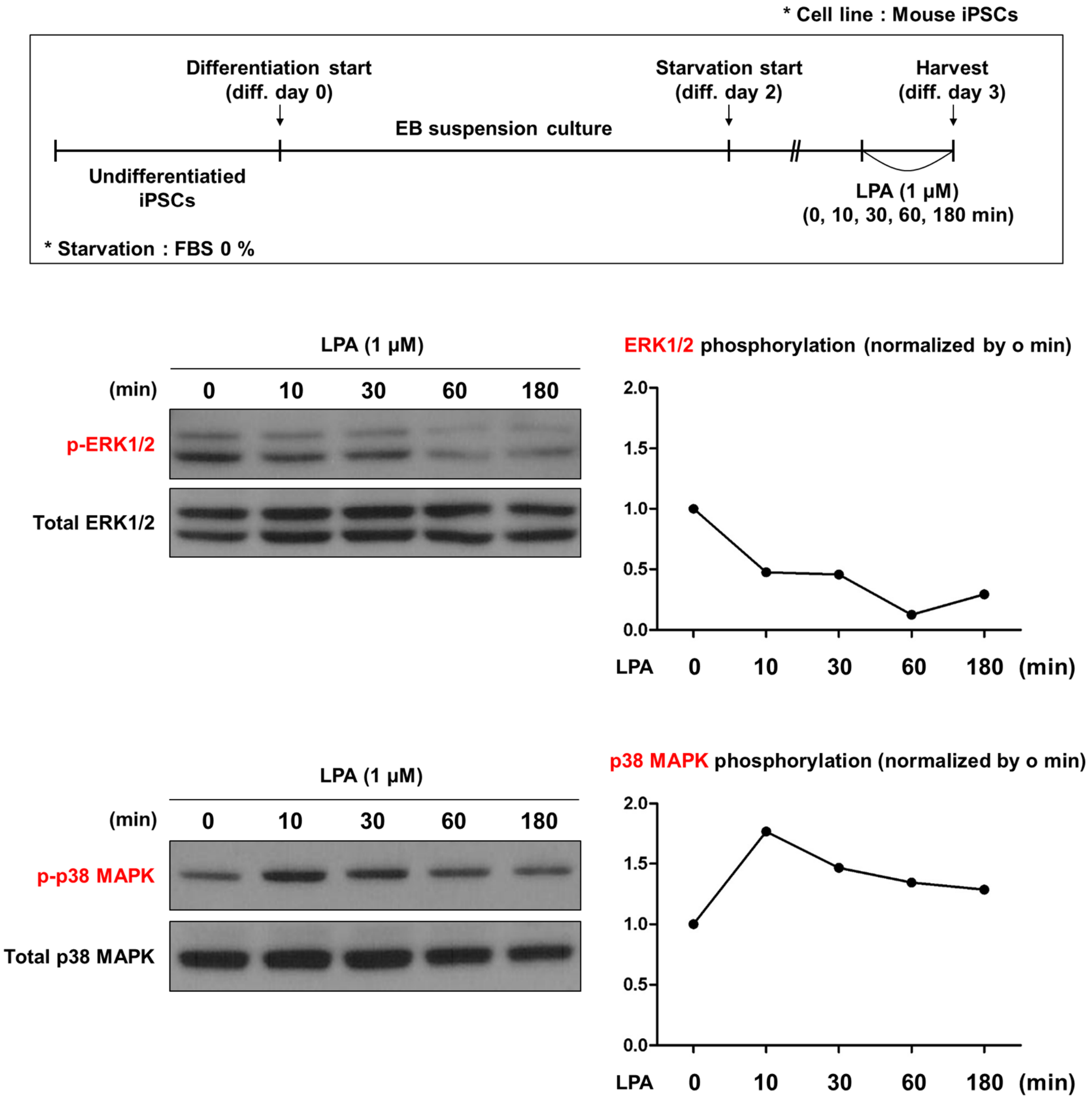

Supplemental Figure 8. Identification of LPAR4 downstream signaling molecules among representative MAPK signals (phosphorylation of ERK1/2 and p38 MAPK).

The effects of LPAR4 stimulation with LPA confirmed by western blotting during the cardiac differentiation on day 3. On cardiac differentiation day 3, LPA was treated with time-point (0 min, 10 min, 30 min, 60 min, and 180 min), respectively, and discovered the signaling molecule that increased with LPA stimulation. The cell line was starved for 1 day and treated with LPA. All experiments were conducted at least in triplicate.

Supplemental Figure 9

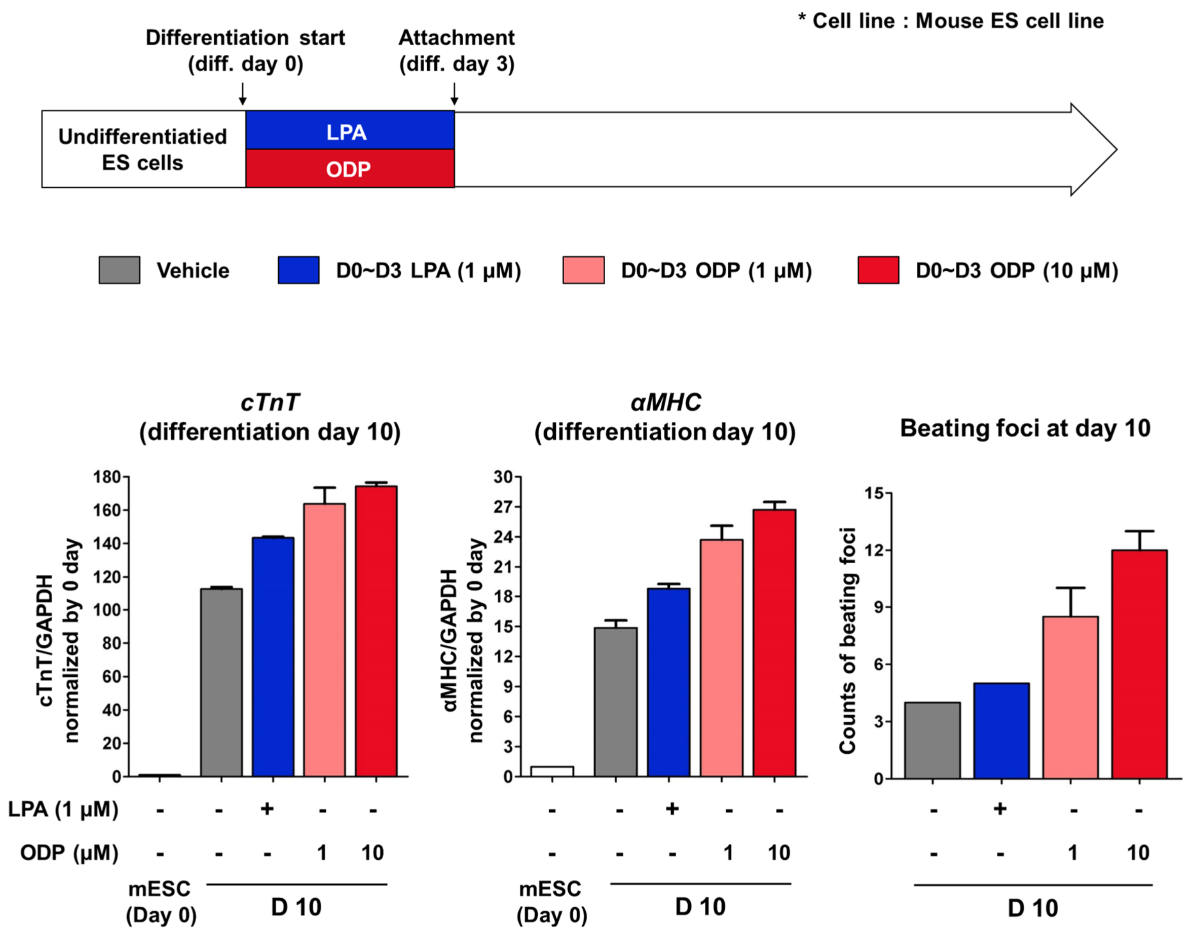

Supplemental Figure 9. Comparison of LPA and ODP (LPAR4 specific agonist) in cardiac differentiation efficiency.

The efficiency of OPD at high concentration was best in inducing mouse ESCs to differentiate into beating cardiomyocytes as well as to express cardiac lineage markers. All experiments were conducted at least in triplicate.

Supplemental Figure 10

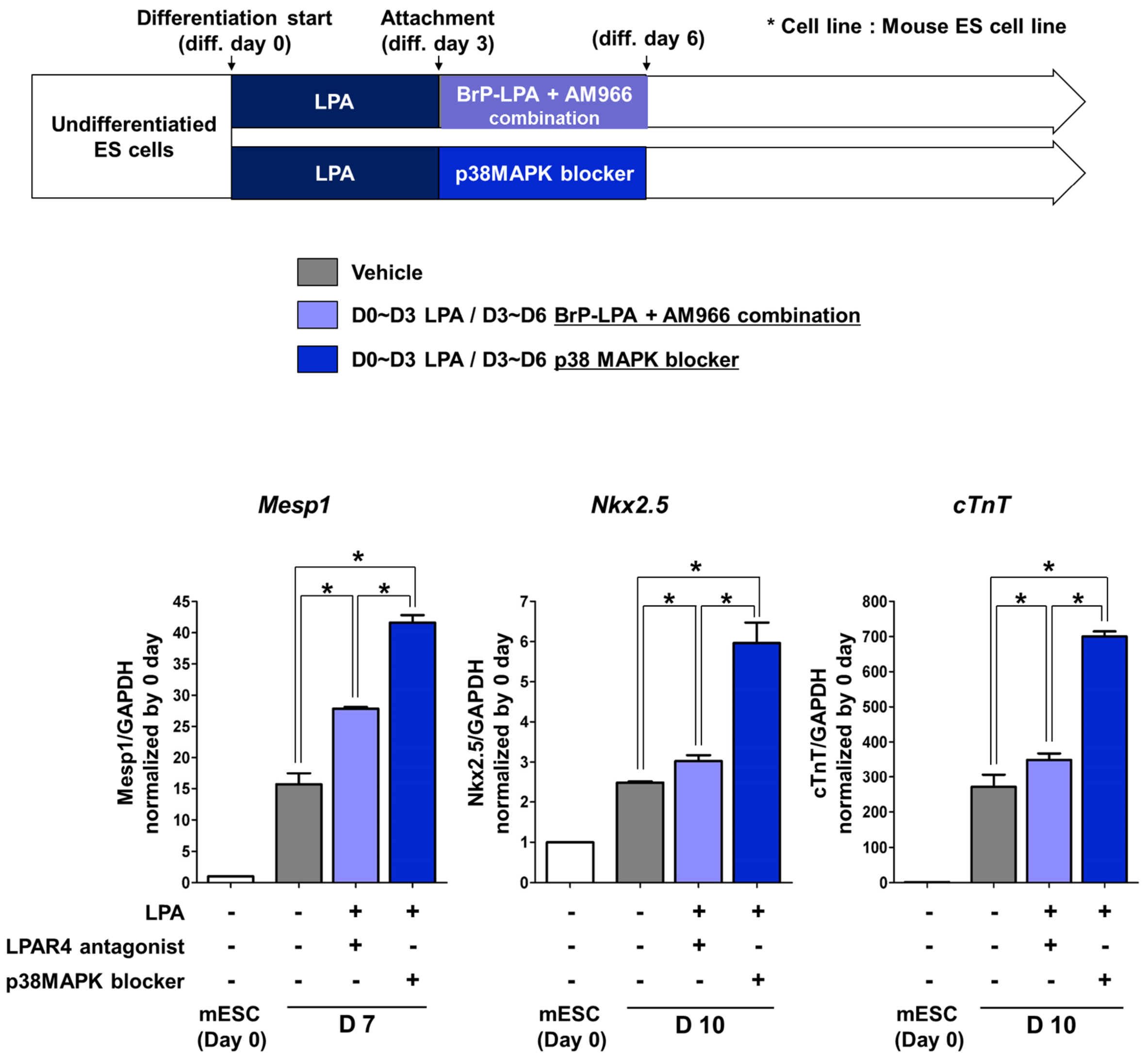

Supplemental Figure 10. Comparison of two kinds of LPAR4 blocker, combination of BrP-LPA/AM966 versus P38MAPK blocker.

The p38 MAPK blocker improves efficiency more than combination of BrP-LPA and AM966 in induction of mouse ESCs to differentiate into cardiac lineage. Statistical analyses were performed using one-way ANOVA (Newman–Keuls). *$P < 0.01$. All experiments were conducted at least in triplicate.

Supplemental Figure 11

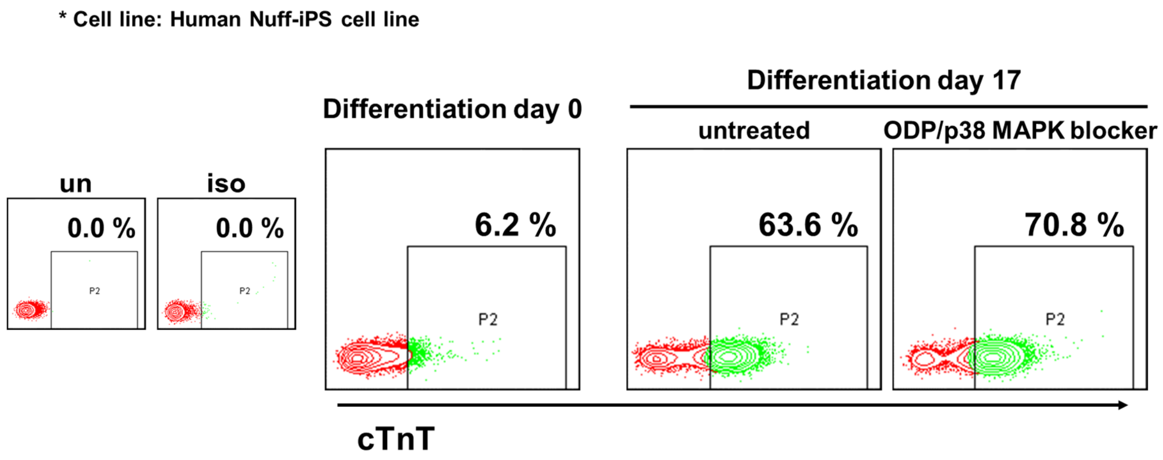

Supplemental Figure 11. Cardiac differentiation protocol using ODP and p38 MAPK blocker in human iPSC.

During the human cardiac differentiation process, the ODP and p38 MAPK blocker (SB203580) were sequentially treated, and the cardiac differentiation efficiency was confirmed by cTnT positivity through FACS analysis.

Supplemental Figure 12

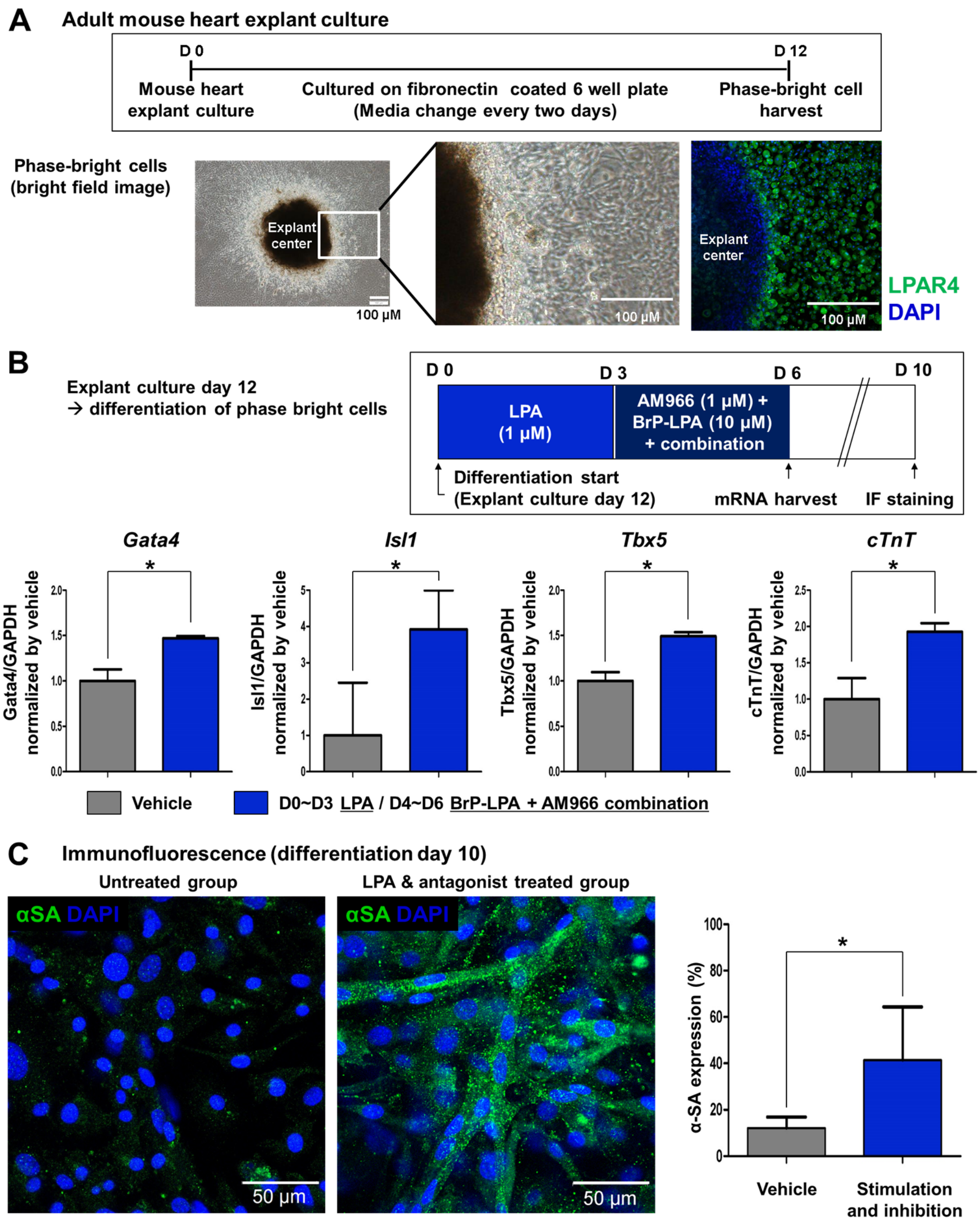

Supplemental Figure 12. Derivation of LPAR4-positive cells from the mouse heart using ex vivo explant-culture.

A Schematic representation of the experimental protocol for 3-week-old mouse heart explant culture and bright-field images of the explant center and expanded cells. The expanded cells were confirmed to be LPAR4-positive by immunofluorescence.

B Schematic diagram of the cardiac differentiation protocol using expanded cells from the explant center. Real-time PCR analysis confirmed the variation in differentiation efficiency between the protocol without treatment and the established cardiac differentiation protocol using the well-known cardiac-related genes, *Gata4*, *Isl1*, *Tbx5*, and *cTnT*.

C Immunofluorescence analysis of αSA; quantitative results are shown in the bar graph. Red, LPAR4; green, αSA; DAPI, nuclei. Bar, 50 μM. Statistical analyses were performed using one-way ANOVA (Newman–Keuls). ***$P < 0.001$. All experiments were conducted at least in triplicate.

Supplemental Figure 13

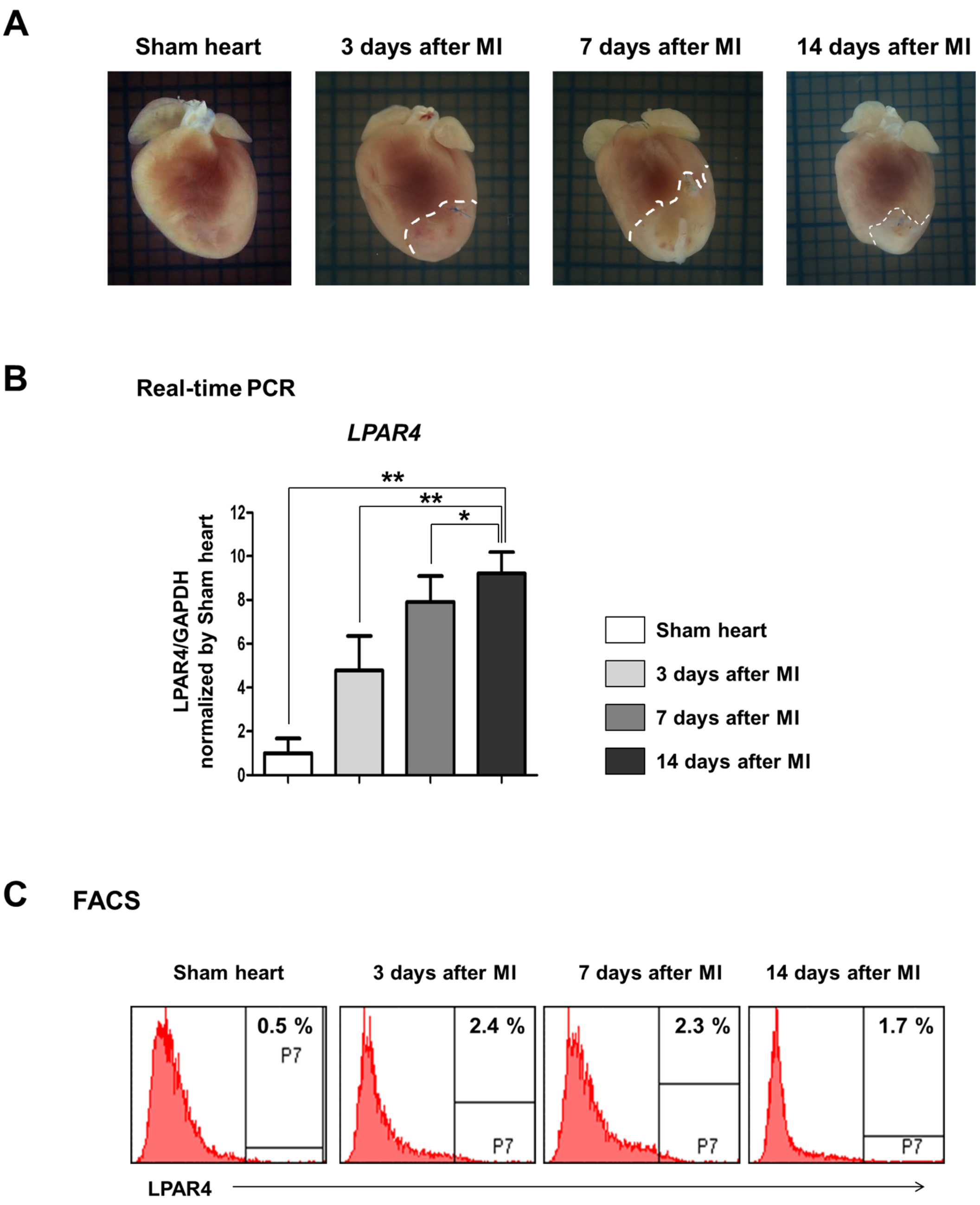

Supplemental Figure 13. Gross images of MI heart and sequential expression pattern of LPAR4 from MI heart.

A Bright-field images of the mouse heart over time after MI. The MI area is indicated by a dotted line.

B Real-time PCR analyses of LPAR4 expression after MI compared with that of the normal mouse heart. The MI heart was harvested at MI progression day 3, day 7, and day 14. Statistical analyses were performed using one-way ANOVA (Newman–Keuls). ***$P < 0.001$. (sham heart, $n = 5$; 3 days after MI, $n = 5$; 7 days after MI, $n = 5$; 14 days after MI, $n = 5$).

C FACS analyses of LPAR4 expression after MI compared with that of the sham heart. The MI heart was harvested at MI progression day 3, day 7, and day 14.

Supplemental Figure 14

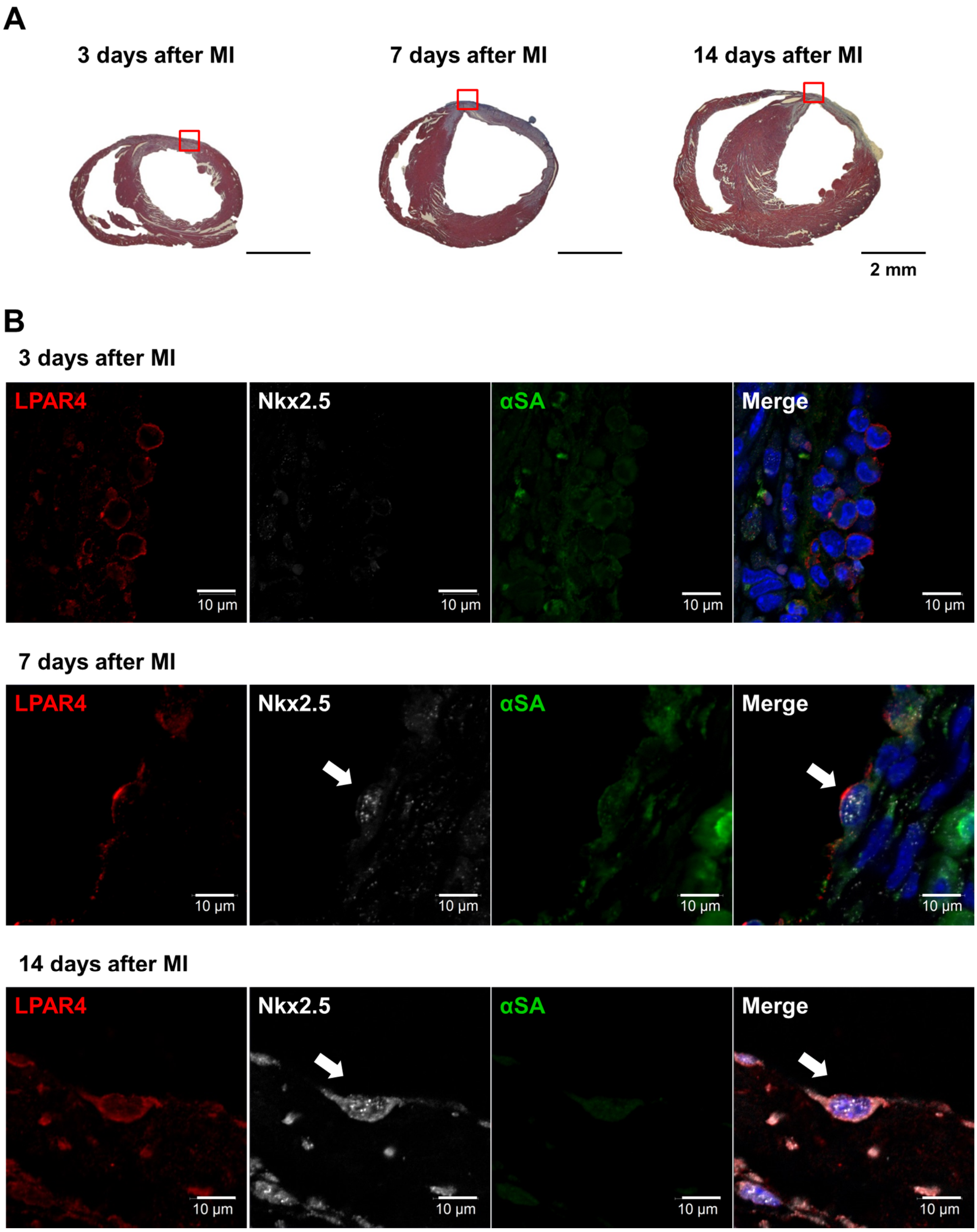

Supplemental Figure 14. The emergence of LPAR4-positive cells at the peri-infarct zone.

A Masson's trichrome staining in the heart after MI.

B Correlation between LPAR4 expression and the expression of well-known cardiac lineage markers (Nkx2.5 and α-SA) in the heart after MI, as analyzed by immunofluorescence. LPAR4 was expressed before Nkx2.5, and Nkx2.5 was expressed in LPAR4-positive cells; αSA was not yet expressed in LPAR4 and Nkx2.5 double-positive cells. Red, LPAR4; white, Nkx2.5; green, αSA; DAPI, nuclei. Bar, 10 µM. All experiments were conducted at least in triplicate.

Supplemental Table 1. Primers used for PCR

Target genes	Sequences
Mouse GAPDH Forward	5’-gaccccttcattgacctcaac-3’
Mouse GAPDH Reverse	5’-cttctccatggtggtgaaga-3’
Mouse Mesp1 Forward	5’-cctgaccaagatcgagacg-3’
Mouse Mesp1 Reverse	5’-acgacaccccgctgcaga-3’
Mouse Nkx2.5 Forward	5’-gacaaagccgagacggatgg-3’
Mouse Nkx2.5 Reverse	5’-ctgtcgcttgcacttgtagc-3’
Mouse Mef2c Forward	5’-gtcagttgggagcttgcacta-3’
Mouse Mef2c Reverse	5’-cggtctctaggaggagaaaca-3’
Mouse cTnT Forward	5’-cagaggaggccaacgtagaag-3’
Mouse cTnT Reverse	5’-ctccatcggggatcttgggt-3’
Mouse αMHC Forward	5’-acggtgaccataaaggagga-3’
Mouse αMHC Reverse	5’-tgtcctcgatcttgtcgaac-3’
Mouse CXCR4 Forward	5’-tcagtggctgacctcctctt-3’
Mouse CXCR4 Reverse	5’-cttggcctttgactgttggt-3’
Mouse RGS5 Forward	5’-attcatccagacagaggccc-3’
Mouse RGS5 Reverse	5’-caagtcaaagctgcgaggag-3’
Mouse LPAR1 Forward	5’-ttctggacccaggaggaatc-3’
Mouse LPAR1 Reverse	5’-acaagaccaatcccggagtc-3’
Mouse LPAR2 Forward	5’-agtctccatcttccccatgc-3’
Mouse LPAR2 Reverse	5’-agcctccctgaatgtttgct-3’
Mouse LPAR3 Forward	5’-tgtgcaataaaaacggctcc-3’
Mouse LPAR3 Reverse	5’-ctcaaacaaccctgtccacg-3’
Mouse LPAR4 Forward	5’-gcttccgcatgaaaatgaga -3’
Mouse LPAR4 Reverse	5’-gtgtcaccaaaaggccagtg-3’
Mouse LPAR5 Forward	5’-ctacagcctggtattggcga-3’
Mouse LPAR5 Reverse	5’-atagcggtccacgttgatga-3’
Mouse LPAR6 Forward	5’-ctgcatcgctgtttccaact-3’
Mouse LPAR6 Reverse	5’-agccggagagatagttccca-3’
Mouse S1PR1 Forward	5’-tttgcactgagccaaaggtc-3’
Mouse S1PR1 Reverse	5’-ggggagacagggtgagaaga-3’
Mouse S1PR2 Forward	5’-tcattcctggaactcctccc-3’
Mouse S1PR2 Reverse	5’-aagttgcaagcagccacatc-3’
Mouse S1PR3 Forward	5’-atgatgtctccctgcgttca-3’
Mouse S1PR3 Reverse	5’-gaacctgggacagcagtgtg-3’
Mouse S1PR4 Forward	5’-acagttggaacagttgggca-3’
Mouse S1PR4 Reverse	5’-tcctgagcaactgtgggtgt-3’
Mouse S1PR5 Forward	5’-tgctttagagcgccacctta-3’
Mouse S1PR5 Reverse	5’-gtcctaagcagttccagccc-3’

Supplemental Methods and Materials

1. Micro-array

In this study, we executed global gene expression analysis using Affymetrix GeneChip® Mouse Gene 2.0 ST Arrays. The sample preparation was performed according to the instructions and recommendations provided by the manufacturer. Total RNA was isolated using RNeasy Mini Kit columns as described by the manufacturer (79645, Qiagen, Hilden, Germany). RNA quality was assessed by Agilent 2100 bioanalyser using the RNA 6000 Nano Chip (Agilent Technologies), and quantity was determined by Nanodrop-1000 Spectrophotometer (Thermo Fisher Scientific). Per RNA sample, 300 ng was used as input into the Affymetrix procedure as recommended by protocol (http://www.affymetrix.com). Briefly, 300 ng of total RNA from each sample was converted to double-strand cDNA Using a random hexamer incorporating a T7 promoter, amplified RNA (cRNA) was generated from the double-stranded cDNA template though an IVT (in-vitro transcription) reaction and purified with the Affymetrix sample cleanup module. cDNA was regenerated through a random-primed reverse transcription using a dNTP mix containing dUTP. The cDNA was then fragmented by UDG and APE 1 restriction endonucleases and end-labeled by terminal transferase reaction incorporating a biotinylated dideoxynucleotide. Fragmented end-labeled cDNA was hybridized to the GeneChip® Human Gene 2.0 ST arrays for 17 hours at 45 °C and 60 rpm as described in the Gene Chip Whole Transcript (WT) Sense Target Labeling Assay Manual (Affymetrix). After hybridization, the chips were stained and washed in a Genechip Fluidics Station 450 (Affymetrix) and scanned by using a Genechip Array scanner 3000 7G (Affymetrix). The expression intensity data were extracted from the scanned images using Affymetrix Command Console software version 1.1 and stored as CEL files. The intensity values of CEL files were normalized to remove bias between the arrays[1], using the Robust Multi-array Average (RMA) algorithm implemented in the Affymetrix Expression Console software (version 1.3.1.) (http://www.affymetrix.com). The whole normalized data were imported into the programming environment R (version 3.0.2) and overall signal distributions of each array were compared by plotting using tools available from the Bioconductor Project (http://www.bioconductor.org)[2] to check good normalization. After confirming whether the data were properly normalized, differentially expressed genes (DEGs) that showed over 2-fold difference between the average signal values of the control groups and treatment groups were selected in manual. In addition, the normalized data of selected DEGs were also imported into the programming environment R for the statistical t-test and genes with p-value less than 0.05 were extracted as significant DEGs for further study[2]. In order to classify the co-expression gene groups which have similar expression patterns, hierarchical clustering analysis

was performed with the MEV (Multi Experiment Viewer) software version 4.4 (http://www.tm4.org)[3]. Finally, using the web-based tool DAVID (the Database for Annotation, Visualization, and Integrated Discovery), DEGs were functionally annotated and classified based on the information of gene function such as OMIMDISEASE, GENE ONTOLOGY, KEGG PATHWAY and BIOCARTA databases to reveal regulatory networks that they are involved in (http://david.abcc.ncifcrf.gov)[4].

2. Mouse cardiomyocyte differentiation

Mouse ESCs (ES-C57BL/6, ATCC® number: SCRC-1002™, ATCC, Manassas, USA) /iPSCs[5] were cultured with mESC media including mouse LIF (recombinant mouse LIF, ESG1107, Merck Millipore, Darmstadt, Germany) on feeder cells, MEF (CF-1, ATCC® number: SCRC-1040™, Manassas, USA). 2,500,000 mouse ESCs/iPSCs were incubated per well of in an aggrewell (#27845/27945, STEMCELL™ technologies, Vancouver, Canada) in embryoid body medium with BMP-4 (recombinant mouse BMP-4, 5020-BP, R&D systems, Minneapolis, USA) for one day to formation embryoid bodies (EBs). And incubated suspension culture for two days in embryoid body medium with BMP-4, Activin A (recombinant human/mouse/rat Activin A, 338-AC, R&D systems, Minneapolis, USA), and bFGF (recombinant human bFGF, 13256029, Thermo Fisher Scientific, Massachusetts, USA) and attached EBs at CMC differentiation day three and media changes every two days. After attaching EBs, medium changes into cardiomyocyte differentiation medium, including bFGF, rhEGF (recombinant human EGF, 236-EG, R&D systems, Minneapolis, USA), rhCT-1 (recombinant human CT-1, 612-CD, R&D systems, Minneapolis, USA), and rmVEGF (recombinant mouse VEGF, 493-MV, R&D systems, Minneapolis, USA) and medium changes every two days.

3. Human cardiomyocyte differentiation

Human iPSCs was reprogramed the NuFF (Newborn Foreskin Fibroblast, GSC-3006G, (Nuff, AMS Biotechnology (GlobalStem), Abingdon, U.K.) with Yamanaka 4 factors. Human iPSCs were cultured with DMEM/F12 Glutamax (10565-018, Thermo Fisher Scientific, Massachusetts, USA) on feeder cells, STO (SIM, ATCC® number: CRL-1503™, Manassas, USA). Human iPSC colonies were detached by dispase (17105-041, Thermo Fisher Scientific, Massachusetts, USA) and dissociated into a single cell and seeded 2,000,000 human iPS cells on matrigel (354277, Corning, New York, USA) coated 35 mm dish. Human iPSCs cultured in 35 mm

dishes are grown on mTeSR™1 (#85851, STEMCELL™ technologies, Vancouver, Canada) until confluence reaches 100%. When human iPSCs confluence reaches 100%, cardiac differentiation progresses sequentially. The order is CHIR99021 (252917-06-9, Cayman, Michigan, USA) for three days, ActivinA (recombinant human/mouse/rat Activin A, 338-AC, R&D systems, Minneapolis, USA) and bFGF (recombinant human bFGF, 13256029, Thermo Fisher Scientific, Massachusetts, USA) for one day, and IWR1 (I0161, Sigma-Aldrich, St. Louis, USA) treated for two days. Then, media change is performed once every two days with human cardiac differentiation media. Human cardiac differentiation media is media supplemented with B27 supplement in RPMI 1640 medium (11875-085, Thermo Fisher Scientific, Massachusetts, USA).

4. Realtime-PCR

All RNAs were separated and purified by cardiomyocyte differentiation and cell harvesting at representative time points. RNeasy® mini kit (74104, QIAGEN, Hilden, Germany) and QIAshredder (79654, QIAGEN, Hilden, Germany) were used to separate and purify RNA from cells. And to synthesis the cDNA from RNA, we used qPCR RT master mix from Toyobo (FSQ-201, TOYOBO, Osaka, Japan). The 7th and 10th days of differentiation were representative times of cardiomyocyte differentiation. The primer sequences are shown at the supplementary table.

5. Flow cytometric analysis and Fluorescence-activated cell sorting analysis

While differentiating mouse ESCs / iPSCs, differentiated cells were dissociated into single cells at flow cytometric analysis and fluorescence-activated cell sorting analysis, incubated with the following antibodies: Flk-1-PE (12-5821-82, Thermo Fisher Scientific, Massachusetts, USA), PDGFRα-APC (17-1401-81, Thermo Fisher Scientific, Massachusetts, USA), CXCR4 (sc-6279, Santa Cruz Biotechnology, Texas, USA), RGS5 (HPA001821, Sigma-Aldrich, St. Louis, USA), LPAR4 (sc-46021, Santa Cruz Biotechnology, Texas, USA), Nkx2.5 (sc-8697, Santa Cruz Biotechnology, Texas, USA), and cTnT (ab10214, Abcam, Cambridge, UK), c-kit (ab24870, Abcam, Cambridge, UK). Flow cytometric analysis were performed using BD FACS Canto™II (Becton Dickinson, New Jersey, USA) and Fluorescence-activated cell sorting analysis were performed using BD FACS Aria™III (Becton Dickinson, New Jersey, USA).

6. Immunofluorescence staining

The cells were plated on confocal dish (ibidi, Freiburg, Germany) and were fixed with 4 % paraformaldehyde and antibodies against: LPAR4 (sc-46021, Santa Cruz Biotechnology, Texas, USA), Oct4 (sc-5279, Santa Cruz Biotechnology, Texas, USA), Nanog (sc-33759, Santa Cruz Biotechnology, Texas, USA), Nkx2.5 (ab91196, Abcam, Cambridge, UK), α-SA (A2172, Sigma-Aldrich, St. Louis, USA).

And mouse heart tissue sections incubated with antibodies against: CXCR4 (sc-6279, Santa Cruz Biotechnology, Texas, USA), RGS5 (HPA001821, Sigma-Aldrich, St. Louis, USA), LPAR4 (sc-46021, Santa Cruz Biotechnology, Texas, USA), α-SA (A2172, Sigma-Aldrich, St. Louis, USA).

At least three different heart MT stained section to quantification of fibrosis area, and we used SABIA software.

7. Western blot

To demonstrate the effect of LPA, we performed western blotting and used antibodies agonist: phospho-p38MAPK (#9211s, Cell Signaling Technology, Massachusetts, USA), total p38MAPK (#9212, Cell Signaling Technology, Massachusetts, USA), phospho-Src (#6943, Cell Signaling Technology, Massachusetts, USA), total Src (#2109, Cell Signaling Technology, Massachusetts, USA), phospho-ERK1/2 (#4370, Cell Signaling Technology, Massachusetts, USA), total ERK1/2 (#9102, Cell Signaling Technology, Massachusetts, USA), phospho-AKT (#9271, Cell Signaling Technology, Massachusetts, USA), total AKT (#9272, Cell Signaling Technology, Massachusetts, USA), Actin (sc-1615, Santa Cruz Biotechnology, Texas, USA).

8. Animals

C57BL/6 wild type mice were used for mouse myocardial infarction model and heart explant culture. C57BL/6 wild type mice were obtained from Orient Bio (Seongnam-si, Republic of Korea) and acclimated 3 to 5 days before challenge. All mice were specified by the supplier to be free of murine viruses, pathogenic bacteria, and endo- and ectoparasites. Mice were housed separately in static cages on aspen bedding. Animals were housed at a temperature of 22 to 24°C with humidity of 40 to 60% and a 12-h light, 12-h dark cycle. We approved by the Institutional Animal Care and Use Committee (IACUC) at Seoul National University Hospital for all animal experiments. Eighty C57BL/6 wild type mice were used in the study.

9. Mouse MI model and echocardiography

C57BL/6 wild type, 7-week-old mice were used for the MI model. The mouse MI model was constructed by tying up the left anterior descending (LAD) artery. Echocardiography values were measured after 14 days of MI in all groups.

Left Ventricular End Systolic Diameter (LVESD) and Left Ventricular End Diastolic Diameter (LVEDD) were measured to determine cardiac function.

Left Ventricular Fractional Shortening (LVFS) was calculated by following formula,

(LVEDD – LVESD)/LVEDD x 100 (%)

Left Ventricular Ejection Fraction (LVEF) was calculated by following formula,

(LVEDD^2 – LVESD^2)/LVEDD^2 x 100 (%)

Immuno-stained MI heart was harvest at day 3, day 7, and day 14 and fixed with 4 % paraformaldehyde (PFA).

10. Mouse heart explant culture

C57BL/6 wild type mice, 3-week-old were used for heart explant culture. Five mice heart were chopped with dissection scissor into similar size, and attached on fibronectin (F0895, Sigma-Aldrich, St. Louis, USA) coated 6 well plate. Do not touch the 6 well plate at least two days because chopped heart fragments attaches slowly. And after two or three days later, medium changes every two days.

11. Statistical analysis

All data are expressed as means ± SEM. The one-way ANOVA analysis of variance using Newman-Keuls' multiple comparison tests was applied to each group comparison using GraphPad Prism 5. P-values < 0.01 were considered statistically significant.

Supplemental References:

1. Irizarry, RA, Hobbs, B, Collin, F, Beazer-Barclay, YD, Antonellis, KJ, Scherf, U*, et al.* (2003). Exploration, normalization, and summaries of high density oligonucleotide array probe level data. *Biostatistics* **4**: 249-264.
2. Gentleman, RC, Carey, VJ, Bates, DM, Bolstad, B, Dettling, M, Dudoit, S*, et al.* (2004). Bioconductor: open software development for computational biology and bioinformatics. *Genome Biol* **5**: R80.
3. Eisen, MB, Spellman, PT, Brown, PO, and Botstein, D (1998). Cluster analysis and display of genome-wide expression patterns. *Proc Natl Acad Sci U S A* **95**: 14863-14868.
4. Sherman, BT, Huang da, W, Tan, Q, Guo, Y, Bour, S, Liu, D*, et al.* (2007). DAVID Knowledgebase: a gene-centered database integrating heterogeneous gene annotation resources to facilitate high-throughput gene functional analysis. *BMC Bioinformatics* **8**: 426.
5. Cho, HJ, Lee, CS, Kwon, YW, Paek, JS, Lee, SH, Hur, J*, et al.* (2010). Induction of pluripotent stem cells from adult somatic cells by protein-based reprogramming without genetic manipulation. *Blood* **116**: 386-395.
